# Synthesis of 2-substituted tryptophans via a C3- to C2-alkyl migration

**DOI:** 10.3762/bjoc.10.207

**Published:** 2014-08-26

**Authors:** Michele Mari, Simone Lucarini, Francesca Bartoccini, Giovanni Piersanti, Gilberto Spadoni

**Affiliations:** 1 Department of Biomolecular Sciences, University of Urbino “Carlo Bo”, Piazza del Rinascimento 6, 61029 Urbino (PU), Italy

**Keywords:** Dehydroalanine, Friedel–Crafts alkylation, indoles, migration, tryptophans

## Abstract

The reaction of 3-substituted indoles with dehydroalanine (Dha) derivatives under Lewis acid-mediated conditions has been investigated. The formation of 2-substituted tryptophans is proposed to occur through a selective alkylative dearomatization–cyclization followed by C3- to C2-alkyl migration and rearomatization.

## Introduction

Facile access to tryptophan and unnatural tryptophan derivatives is of general interest because tryptophans are found in many naturally occurring compounds and are an important component of biologically active compounds [1–7]. Tryptophan and tryptophan analogs also have applications in chemical biology thanks to the highly environment-sensitive fluorescence properties of the indole ring [8–17] and when incorporated into peptides, they lead to compounds with increased resistance to enzymatic degradation and modification [18–25].

As part of our ongoing research on the use of the unsaturated amino acid dehydroalanine (Dha) in organic synthesis [26–37], we have focused our attention on the syntheses of tryptophans, cyclo-tryptophans (also known as pyrroloindolines), and tryptophan-containing natural products from simple indole starting materials [38–44].

**Scheme 1:**
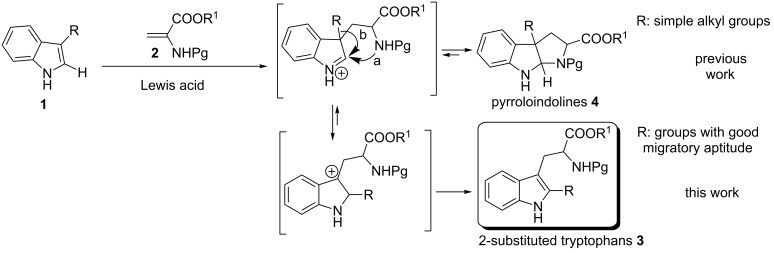
Friedel–Crafts alkylation of 3-substituted indoles.

In 2010, we reported a novel one-pot approach for the preparation of pyrroloindolines **4** by a cascade addition/cyclization strategy between simple alkyl C3-substituted indoles **1** and 2-amidoacrylates **2** in the presence of stoichiometric amounts of a hard Lewis acid (Scheme 1, path a) [39]. Good yields and high exo:endo diastereoselectivities were obtained for a variety of indoles.

If the reaction is performed with indoles containing groups with good migratory aptitude at the C3 position, a mixture of the expected pyrroloindoline **4** and 2-substituted tryptophans **3** was observed. A plausible mechanism for the formation of this unexpected side product involves rearrangement of pyrroloindoline **4** to the corresponding 2-substituted tryptophan **3** by a C3- to C2-alkyl indole migration and rearomatization (Scheme 1, path b). Therefore, the aim of this study was to exploit the C3 Friedel–Crafts (FC) alkylation/C3- to C2 alkyl migration sequence for the synthesis of 2-substituted tryptophans [45–50].

The known methods for the synthesis of 2-substituted tryptophans are limited and include both the catalytic and non-catalytic union of 2-alkylindole with a protected aziridine-2-carboxylate or an α-aminoenoate [38,51–54] or directly from 2-unsubstituted protected tryptophan and the appropriate nucleophiles via a 3-chloroindolenine intermediate [55–57]. More recently, direct C2-arylation and alkylation of *N*-protected tryptophan methyl ester have been reported in the context of a more extensive study on C−H activation reactions [58–61]. The present procedure is comparable to those described previously in terms of yield, but it is superior to previous methods with respect to its simplicity as it employs easily accessible 3-substituted indoles.

## Results and Discussion

Initially, the optimal conditions for the critical alkylative dearomatization–cyclization followed by the migration/rearomatization reaction process were explored. Our initial attempt involved reacting readily available 3-benzylindole (**1a**) with commercially available *N*-acetyl-dehydroalanine methyl ester (**2a**) under the reaction conditions previously optimized for the synthesis of pyrroloindolines. However, in the presence of ZrCl_4_ (2 equiv), the reaction gave a low conversion (Table 1, entry 1).

It was found that the non-coordinating solvent CH_2_Cl_2_ gave the best results whereas moving to more polar solvents such as ethanol, DMF, and THF proved to be detrimental, presumably due to coordination to the Lewis acid (Table 1, entries 2–4). The use of a strong H-bond donor such as trifluoroethanol (TFE) did not accelerate the reaction and still gave low yields after 24 h (Table 1, entry 5). Next, we tested the effect of different acids on the reaction. Although some Lewis acids such as TiCl_4_, SnCl_4_, and Sc(OTf)_3_ did not show beneficial effects (Table 1, entries 6–8), a good yield of 2-benzyltryptophan was achieved when 2 equiv of EtAlCl_2_ was used (Table 1, entry 9). Notably, resubmission of isolated pyrroloindoline **4a**, obtained by reducing the reaction time to five hours (Table 1, entry 12), to the exact reaction conditions above, provided another batch of 2-benzyltryptophan (**3a**), showing that pyrroloindoline is the intermediate of the reaction. However, increasing the amount of acid did not afford a higher yield (Table 1, entry 11); but on the contrary, a smaller amount prevented the reaction from going to completion (Table 1, entry 10). Despite research by Jackson et al*.* [45–50] showing an intramolecular rearrangement to yield 2,3-disubstituted indoles using TFA or diluted HCl, our synthetic procedure did not work with the addition of these acids, and only some indole oligomers were obtained. The best yield and reactivity were obtained by conducting the reaction with 2 equiv of EtAlCl_2_ in CH_2_Cl_2_ at room temperature for 24 hours (Table 1, entry 9).

**Table 1:** Optimization of the reaction conditions.^a^

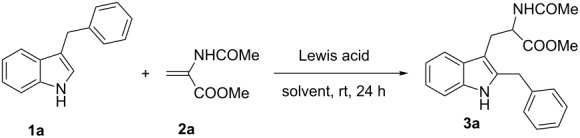			

Entry	Lewis acid	Solvent	Yield (%)^b^

1	ZrCl_4_	CH_2_Cl_2_	25
2	ZrCl_4_	EtOH	NR
3	ZrCl_4_	DMF	NR
4	ZrCl_4_	THF	NR
5	ZrCl_4_	TFE	13
6	TiCl_4_	CH_2_Cl_2_	15
7	SnCl_4_	CH_2_Cl_2_	28
8	Sc(OTf)_3_	CH_2_Cl_2_	12
9	EtAlCl_2_	CH_2_Cl_2_	70
10^c^	EtAlCl_2_	CH_2_Cl_2_	29
11^d^	EtAlCl_2_	CH_2_Cl_2_	68
12^e^	EtAlCl_2_	CH_2_Cl_2_	25

^a^Reaction conditions: **1a** (0.25 mmol), **2a** (0.3 mmol), Lewis acid (0.5 mmol), solvent (2.5 mL), rt, 24 h. ^b^Yields of the isolated products after column chromatography. ^c^Lewis acid (0.25 mmol). ^d^Lewis acid (1 mmol). ^e^The reaction was quenched after 5 hours to isolate the pyrroloindolines **4a**, see Supporting Information File 1. NR, no reaction.

Under the optimized reaction conditions (Table 1, entry 9), the substrate scope was then examined, focusing on the relative migratory aptitudes of various C3-indole substituents; the results are summarized in Table 2. The reaction worked well, affording good to excellent yields using 3-(*p*-methoxybenzyl)indole (**1b**) and 3-(*p*-chlorobenzyl)indole (**1c**), whereas it did not afford the desired 2-substituted tryptophan when 3-(*p*-nitrobenzyl)indole (**1d**) was used as the starting material. These results can be attributed to the greater migratory aptitude of both the *p*-methoxy- and *p*-chlorobenzyl groups, compared to the *p*-nitrobenzyl substituent (even though in this case a detrimental coordination between nitro group and Lewis acid can occur), thus agreeing with earlier studies on the benzylation of indoles [46].

Whereas indoles bearing a simple C3-benzyl substituent furnish products in good yields, the reaction is very sensitive to the steric bulk around the electrophilic alkyl carbon atom; this observation is in agreement with the fact that the C3-alkyl group is very likely to attack the electrophilic iminium species generated in situ after the Dha/Friedel–Crafts-type reaction with indoles. Attempts to carry out the alkylation/migration reaction with 3-benzhydrylindole (**1e**) were unfruitful (Table 2, entry 5). When 3-(tetrahydronaphthalen-1-yl)indole (**1f**) was used, a very low conversion to the corresponding 2-substituted tryptophan was observed (Table 2, entry 6). For indoles containing 3-heterobenzyl substituents, the results were conflicting. Whereas 3-(furan-2-ylmethyl)indole (**1g**) did not react under the usual reaction conditions (Table 2, entry 7), bis(indol-3-yl)methane (**1h**) provided the desired product in an excellent yield and decent time (Table 2, entry 8). The latter is an interesting compound, and to the best of our knowledge, it has never been synthesized previously but only reported as a contaminant in biotechnologically manufactured tryptophan [62–63]. Also 3-benzyl-*N*-methylindole (**1i**) performed well in the reaction although a longer reaction time is needed to obtain a reasonable yield of the desired **3i** (Table 2, entry 9). Regarding on the influence of the amine protecting group of Dha, the *N*-acetyl protecting group might promote the C3–C2 rearrangement, rendering the intermediate 3,3-disubstituted indolenium salt more likely to accept the migrating alkyl group at the C2-position that is in equilibrium with the corresponding tricyclic pyrroloindolines. Indeed, the reaction between 3-benzylindole (**1a**) and methyl 2-phthalimidoacrylate (**2b**) gave only 48% yield of the desired rearranged product (**3j**) (Table 2, entry 10) whereas the *N*-Cbz and *N*-Boc protecting groups were unstable under the reaction conditions.

**Table 2:** Synthesis of 2-benzyltryptophans **3a–j**.^a^

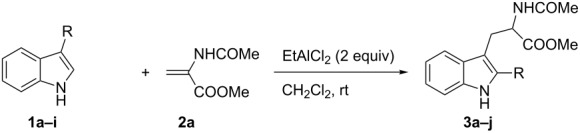				

Entry	Indole	Tryptophan	Time (h)	Yield (%)^b^

1	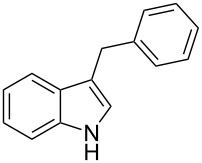 **1a**	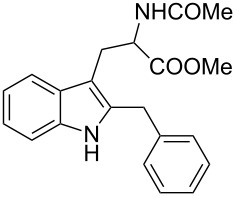 **3a**	24	70
2	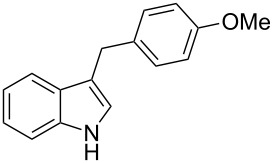 **1b**	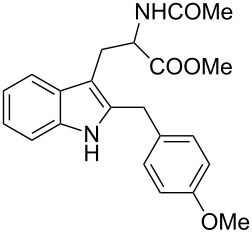 **3b**	16	74
3	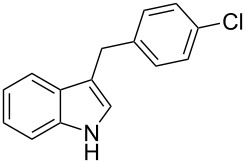 **1c**	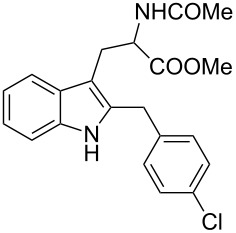 **3c**	48	53
4	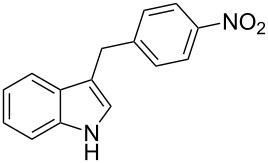 **1d**	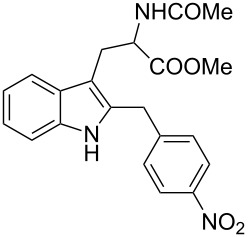 **3d**	72	NR
5	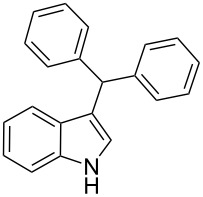 **1e**	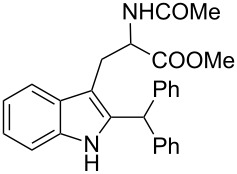 **3e**	72	NR
6	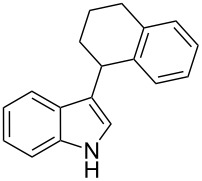 **1f**	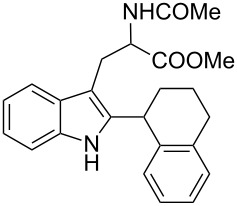 **3f**	72	11
7	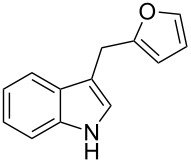 **1g**	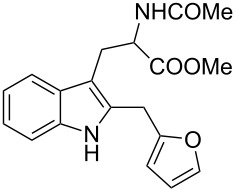 **3g**	72	NR
8	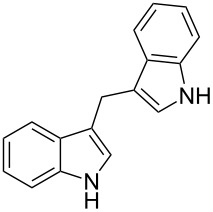 **1h**	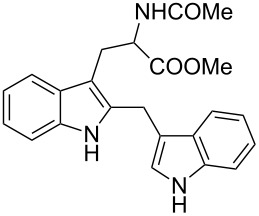 **3h**	24	67
9	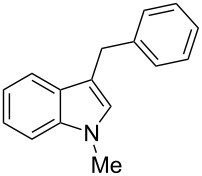 **1i**	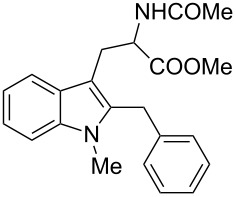 **3i**	48	51
10^c^	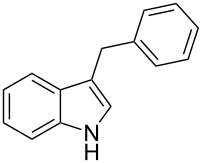 **1a**	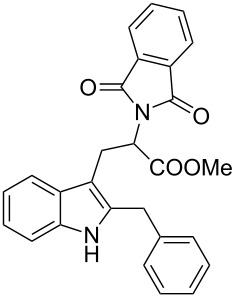 **3j**	24	48

^a^Reaction Conditions: **1a–i** (0.25 mmol), **2a** (0.3 mmol), EtAlCl_2_ (0.5 mmol), CH_2_Cl_2_ (2.5 mL), rt. ^b^Yields of the isolated products after column chromatography. ^c^Methyl 2-phthalimidoacrylate (**2b**) was used. NR, no reaction.

As shown in Table 3, this novel reaction with Dha **2a** is not restricted to 3-benzylindole derivatives but can also be employed for other types of S_N_1-active substrates such as 3-allylindoles [64]. Under the optimized conditions, the use of 3-allyl, 3,3-dimethylallyl (“normal” prenyl), and 3-geranylindoles as nucleophiles provided the corresponding 2-allyltryptophans [65] in good yields, after the expected C3- to C2-indole allyl migration (Table 3, entries 1–3). The high yielding synthesis of these compounds is of particular interest as 2-prenyltryptophan derivatives have been obtained or isolated from a diverse array of natural sources [66–67] and, in general, prenylation at the indole ring leads to a significant increase in the antioxidant and/or cytotoxic activity of tryptophan-containing molecules [68–70]. However, the reaction did not occur with the indole bearing the more bulky 1,1-dimethylallyl (“reverse” prenyl) substituent at C3, confirming the limit of steric hindrance in this reaction (Table 3, entry 4). Unfortunately, the reaction with 3-propargylindole only afforded the corresponding pyrrolindoline derivative without any trace of the rearranged 2-substituted tryptophan, even with extended reaction times.

**Table 3:** Synthesis of 2-allyltryptophans **3k–o**.^a^

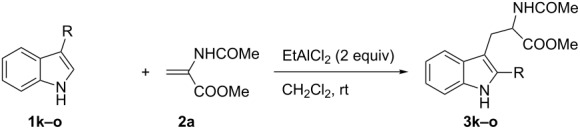				

Entry	Indole	Tryptophan	Time (h)	Yield (%)^b^

1	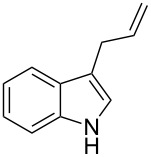 **1k**	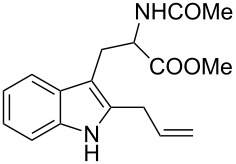 **3k**	48	61
2	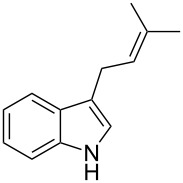 **1l**	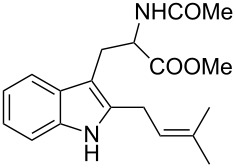 **3l**	16	86
3	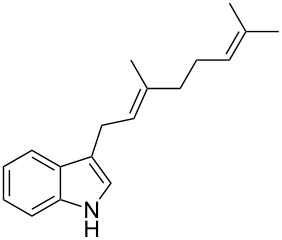 **1m**	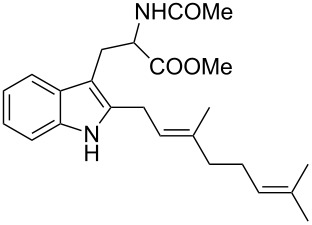 **3m**	16	70
4	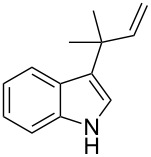 **1n**	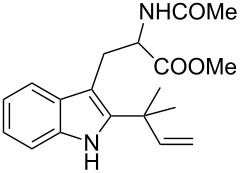 **3n**	72	NR
5	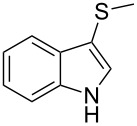 **1o**	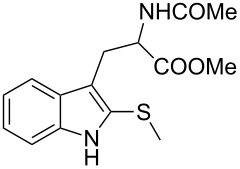 **3o**	48	68

^a^Reaction conditions: **1k–o** (0.25 mmol), **2a** (0.3 mmol), EtAlCl_2_ (0.5 mmol), CH_2_Cl_2_ (2.5 mL), rt. ^b^Yields of the isolated products after column chromatography. NR, no reaction.

The electronic properties of the migratory group have a pronounced effect on the reaction profile. As known from other Wagner–Meerwein-type rearrangements [71], the migration tendency is principally controlled by the stability of the migratory cation. However, we reasoned that indoles with an electron-rich and polarizable atom/functional group at the C3-position (i.e., 3-sulfenylindoles) could be good substrates for the reaction. Notably, 3-(methylthio)indole (**1o**) underwent the alkylation/C3–C2 migration sequence to give 2-(methylthio)tryptophan **3o** in good yields (Table 3, entry 5). Remarkably, the presence of a thioether in the indole ring offers unique, site-specific handles that can be utilized for further functionalization of the tryptophan moiety.

## Conclusion

In summary, we have developed the synthesis of 2-functionalized/substituted tryptophans through a novel alkylative dearomatization–cyclization/migration/rearomatization sequence between easily accessible 3-substituted indoles and commercially available Dha **2a** for the construction of 2-substituted tryptophans. The final rearrangement proceeded in moderate to very good yields, depending on the migration tendencies of the C3-indole substituent. Although the substituent migration from the C3- to C2-indole position is principally limited to benzyl, allyl/prenyl, and sulfenyl groups, the operational simplicity, synthetic brevity, and relatively facile access to 3-substituted indoles should make it very useful for the preparation of C2-functionalized tryptophan derivatives.

## Experimental

### General procedure for the synthesis of *N*-acetyl-2-substituted tryptophan methyl ester

A 1 M solution of EtAlCl_2_ in hexane (2 mmol, 2 mL) was added dropwise to a stirred and cooled (0 °C) mixture of methyl 2-acetamidoacrylate (172 mg, 1.2 mmol) and the suitable 3-substituted indole (1 mmol) in dry CH_2_Cl_2_ (10 mL) under a nitrogen atmosphere. The mixture was stirred at room temperature for 17–72 hours, then carefully poured into an ice-cold saturated aqueous sodium hydrogen carbonate solution (10 mL). The resulting suspension was filtered through Celite and the aqueous layer was extracted with dichloromethane (3 × 15 mL). The combined organic layers were dried over sodium sulfate, filtered and concentrated in vacuo. The crude product was purified by flash chromatography on silica gel (cyclohexane/ethyl acetate 8:2, or CH_2_Cl_2_/methanol 98:2 for **3h**, as eluent) and/or crystallization.

## Supporting Information

File 1 Experimental procedures, characterization data, ^1^H and ^13^C NMR spectra of new compounds.
