# Supplementary material for: Synthesis of 2-substituted tryptophans via a C3- to C2-alkyl migration
Source: Beilstein J Org Chem. 2014 Aug 26;10:1991–8. doi: 10.3762/bjoc.10.207 (PMC4168769; doi:10.3762/bjoc.10.207)
Supplement: File 1 — Experimental procedures, characterization data, 1H and 13C NMR spectra of new compounds. [file Beilstein_J_Org_Chem-10-1991-s001.pdf]

Supporting Information

for

Synthesis of 2-substituted tryptophans via a C3- to C2-alkyl migration

Michele Mari, Simone Lucarini, Francesca Bartoccini, Giovanni Piersanti*,§ and Gilberto Spadoni

Address: Department of Biomolecular Sciences, University of Urbino “Carlo Bo”, Piazza del Rinascimento 6, 61029 Urbino (PU), Italy.

Email: Giovanni Piersanti* - giovanni.piersanti@uniurb.it

*Corresponding author

§Phone, +390722303320; fax, +390722303313

Experimental Procedures and NMR Spectra

Contents

- Materials and Methods S2
- Compounds Characterization and Synthetic Methods S3
- COSY and NOESY Spectra of **3a** S9
- References S11
- ^{1}H and ^{13}C NMR Spectra S12

Materials and Methods

All reactions were run in air unless otherwise noted. Column chromatography purifications were performed in flash conditions using Merck 230–400 mesh silica gel. Analytical thin layer chromatography (TLC) was carried out on Merck silica gel plates (silica gel 60 F_{254}), that were visualized by exposure to ultraviolet light and an aqueous solution of p-anisaldehyde. ^{1}H NMR and ^{13}C NMR spectra were recorded on a Bruker Avance 200 spectrometer, using $CDCl_3$ as a solvent. Chemical shifts (δ scale) are reported in parts per million (ppm) relative to the central peak of the solvent. Coupling constants (J values) are given in Hertz (Hz). ESI-MS spectra were taken on a Waters Micromass ZQ instrument, only molecular ions (M + 1 or M − 1) are given. IR spectra were obtained on a Nicolet Avatar 360 FT-IR spectrometer, absorbance values are reported in cm^{-1}. Melting points were determined on a Buchi SMP-510 capillary melting point apparatus and are uncorrected. Elemental analyses were performed on a Carlo Erba analyzer and the results are within ±0.3 of the theoretical values (C,H,N). Methyl 2-acetamidoacrylate (**2a**) and bis(1*H*-indol-3-yl)methane (**1h**) are commercially available. Starting materials 3-benzyl-1*H*-indole (**1a**)[1], 3-(4-methoxybenzyl)-1*H*-indole (**1b**)[2], 3-(4-chlorobenzyl)-1*H*-indole (**1c**)[2], 3-(4-nitrobenzyl)-1*H*-indole (**1d**)[3], 3-benzhydryl-1*H*-indole (**1e**)[4], 3-((furan-2-yl)methyl)-1*H*-indole (**1g**)[2], 3-benzyl-1-methyl-1*H*-indole (**1i**)[3], 3-allyl-1*H*-indole (**1k**)[5], 3-(3-methylbut-2-enyl)-1*H*-indole (**1l**)[6], 3-((E)-3,7-dimethylocta-2,6-dienyl)-1*H*-indole (**1m**)[6], 3-(2-methylbut-3-en-2-yl)-1*H*-indole (**1n**)[5], 3-(methylthio)-1*H*-indole (**1o**)[7] and methyl 2-(1,3-dioxoisoindolin-2-yl)acrylate (**2b**)[8] were prepared as previously described.

Compounds Characterization and Synthetic Methods

3-(1,2,3,4-tetrahydronaphthalen-1-yl)-1*H*-indole (1f)

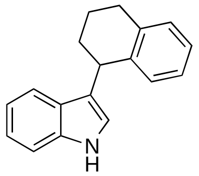

Compound **1f** was prepared according to the procedure reported in literature.[9]
Colorless Oil, TLC: *Rf* = 0.47 (cyclohexane/ethyl acetate, 9:1; UV, p-anisaldehyde). FTIR (nujol): $\tilde{\nu}$ max = 3417 cm^{-1}. ^{1}H NMR (200 MHz, $CDCl_3$): δ = 1.76-2.04 (m, 2H), 2.16-2.25 (m, 2H), 2.84-3.09 (m, 2H), 4.48-4.54 (m, 1H), 6.67 (d, *J*=2.5 Hz, 1H), 7.08-7.30 (m, 6H), 7.37-7.42 (m, 1H), 7.57-7.61 (m, 1H), 7.85 (br s, 1H) ppm. ^{13}C NMR (50 MHz, $CDCl_3$): δ = 20.8, 29.8, 30.9, 36.5, 111.3, 119.2, 119.5, 121.9, 122.3, 123.1, 125.6, 125.8, 126.7, 129.0, 130.1, 136.6, 137.4, 139.7 ppm. MS (ESI): *m/z* (%) = 246 [M-H]$^-$. $C_{18}H_{17}N$ (247.14): calcd. C 87.41, H 6.93, N 5.66; found C 87.31, H 6.98, N 5.69.

Methyl 2-acetamido-3-(2-benzyl-1*H*-indol-3-yl)propanoate (3a)

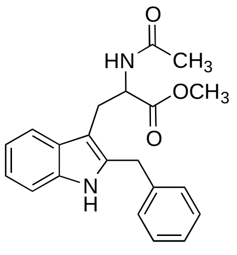

White solid (245 mg, 70%). mp: 168-169 °C (from ether-hexane); TLC: *Rf* = 0.26 (cyclohexane/ethyl acetate, 6:4; UV, p-anisaldehyde). FTIR (nujol): $\tilde{\nu}$ max = 3398, 3397, 1740, 1655 cm^{-1}. ^{1}H NMR (200 MHz, $CDCl_3$): δ = 1.78 (s, 3H), 3,36 (d, *J*=5.5 Hz, 2H), 3.65 (s, 3H), 4.08 (s, 2H), 4.93 (ddd, J_1=J_2=5.5 Hz, J_3=8.0 Hz, 1H), 6.09 (br d, *J*=8.0 Hz, 1H), 7.06-7.38 (m, 8H), 7.47-7.53 (m, 1H), 8.22 (br s, 1H). ^{13}C NMR (50 MHz, $CDCl_3$): δ = 23.0, 26.9, 32.0, 52.4, 53.1, 106.6, 110.8, 118.2, 119.6, 121.7, 126.8, 128.6, 128.7, 128.9, 135.0, 135.6, 138.5, 169.9, 172.6. MS (ESI): *m/z* (%) = 351 [M+H]$^+$; 349 [M-H]$^-$. $C_{21}H_{22}N_2O_3$ (350.16): calcd. C 71.98, H 6.33, N 7.99; found C 72.06, H 6.31, N 7.94.

Methyl 3-(2-(4-methoxybenzyl)-1*H*-indol-3-yl)-2-acetamidopropanoate (3b)

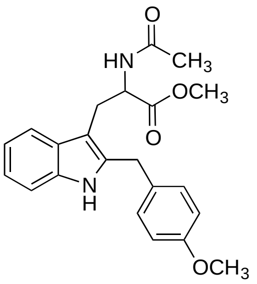

Brown solid (281 mg, 74%). mp: 108-110 °C (from ether-hexane); TLC: *Rf* = 0.24 (cyclohexane/ethyl acetate, 1:1; UV, p-anisaldehyde). FTIR (nujol): $\tilde{\nu}$ max = 3397, 3293, 1736, 1655 cm^{-1}. ^{1}H NMR (200 MHz, $CDCl_3$): δ = 1.83 (s, 3H), 3.35 (d, *J*=5.5 Hz, 2H), 3.65 (s, 3H), 3.79

(s, 3H), 4.01 (s, 2H), 4.93 (ddd, J_1=J_2=5.5 Hz, J_3=8.0 Hz, 1H), 6.04 (d, J=8.0 Hz, 1H), 6.84-6.89 (m, 2H), 7.08-7.24 (m, 5H), 7.46-7.51 (m, 1H), 7.94 (br s, 1H). ^{13}C NMR (50 MHz, $CDCl_3$): δ = 23.1, 26.9, 31.2, 52.5, 53.1, 55.3, 106.4, 110.6, 114.3, 118.1, 119.6, 121.6, 128.8, 129.7, 130.2, 135.4, 135.5, 158.5, 169.7, 172.6. MS (ESI): *m/z* (%) = 381 $[M+H]^+$; 379 $[M-H]^-$. $C_{22}H_{24}N_2O_4$ (380.17): calcd. C 69.46, H 6.36, N 7.36; found C 69.35, H 6.47, N 7.37.

Methyl 3-(2-(4-chlorobenzyl)-1*H*-indol-3-yl)-2-acetamidopropanoate (3c)

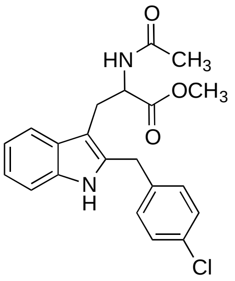

Brown solid (204 mg, 53%). mp: 89-90 °C (from ether-hexane); TLC: *Rf* = 0.31 (cyclohexane/ethyl acetate, 1:1; UV, p-anisaldehyde). FTIR (nujol): $\tilde{\nu}$max = 3383, 3292, 1737, 1657 cm^{-1}. ^{1}H NMR (200 MHz, $CDCl_3$): δ = 1.87 (s, 3H), 3.35 (d, J=5.5 Hz, 2H), 3.64 (s, 3H), 4.06 (s, 2H), 4.93 (ddd, J_1=J_2= 5.5 Hz, J_3= 8.0 Hz, 1H), 6.03 (d, J=8.0 Hz, 1H), 7.07-7.16 (m, 4H), 7.22-7.32 (m, 3H), 7.47-7.52 (m, 1H), 7.85 (br s, 1H). ^{13}C NMR (50 MHz, $CDCl_3$): δ = 23.1, 26.9, 31.4, 52.5, 53.1, 107.0, 110.7, 118.2, 119.8, 121.9, 128.7, 129.0, 129.9, 132.7, 134.3, 135.6, 136.8, 169.7, 172.5. MS (ESI): *m/z* (%) = 385 $[M+H]^+$; 383 $[M-H]^-$. $C_{21}H_{21}ClN_2O_3$ (384.12): calcd. C 65.54, H 5.50, N 7.28; found C 65.45, H 5.42, N 7.37.

Methyl 2-acetamido-3-(2-(1,2,3,4-tetrahydronaphthalen-1-yl)-1*H*-indol-3-yl)propanoate (3f)

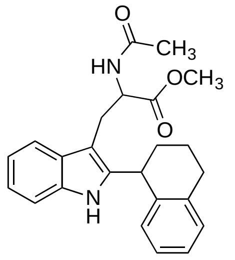

Brown solid (43 mg, 11%). mp: 153-154 °C (from ether-hexane); TLC: *Rf* = 0.27 (cyclohexane/ethyl acetate, 1:1; UV, p-anisaldehyde). FTIR (nujol): $\tilde{\nu}$max = 3351, 3281, 1735, 1655 cm^{-1}. ^{1}H NMR (200 MHz, $CDCl_3$): δ = 1.80-1.94 (m, 2H+2H), 1.90 (s, 3H), 2.01-2.19 (m, 2H+2H), 2.04 (s, 3H), 2.88-3.08 (m, 2H+2H), 3.11-3.43 (m, 2H+2H), 3.75 (s, 3H), 3.77 (s, 3H), 4.16-4.29 (m, 1H+1H), 4.85-4.99 (m, 1H+1H), 6.12 (d, J=7.5, 1H), 6.22 (d, J=7.5, 1H), 6.80-7.16 (m, 10H), 7.42-7.62 (m, 4H), 7.81-7.85 (m, 2H), 8.58 (br s, 1H+1H). ^{13}C NMR (50 MHz, $CDCl_3$): δ = 23.0, 23.1, 23.2, 23.3, 29.2, 29.4, 30.0, 30.1, 31.9, 32.0, 36.6, 36.7, 52.1, 52.2, 52.9, 52.9, 110.8, 110.8, 118.2, 118.2, 118.9, 118.9, 120.0, 120.2, 121.5, 121.5, 125.7, 125.8, 126.6, 126.8, 128.3, 128.3, 128.7, 128.8, 128.9, 129.0, 130.1, 130.1, 136.1, 136.2, 137.0, 137.3, 139.3, 139.7, 170.2, 170.2, 172.4, 172.5. MS (ESI): *m/z* (%) = 391 $[M+H]^+$; 389 $[M-H]^-$. $C_{24}H_{26}N_2O_3$ (390.19): calcd. C 73.82, H 6.71, N 7.17; found C 73.94, H 6.80, N 7.10.

Methyl 3-(2-((1*H*-indol-3-yl)methyl)-1*H*-indol-3-yl)-2-acetamidopropanoate (3h)

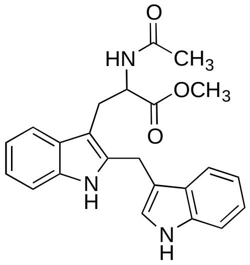

Brown solid (261 mg, 67%). mp: 145-146 °C (from ether-hexane); TLC: *Rf* = 0.24 (cyclohexane/ethyl acetate, 1:1; UV, p-anisaldehyde). FTIR (nujol): $\tilde{\nu}$max = 3398, 3277, 1735, 1655 cm^{-1}. ^{1}H NMR (200 MHz, $CDCl_3$): δ = 1.71 (s, 3H), 3.41 (d, *J*=5.5 Hz, 2H), 3.64 (s, 3H), 4.17 (s, 2H), 4.96 (ddd, J_1=J_2= 5.5 Hz, J_3= 8.0 Hz, 1H), 6.05 (d, *J*=8.0 Hz, 1H), 6.92 (d, *J*=2.5 Hz, 1H), 7.07-7.26 (m, 5H), 7.36-7.52 (m, 3H), 8.01 (br s, 1H), 8.26 (br s, 1H). ^{13}C NMR (50 MHz, $CDCl_3$): δ = 22.3, 22.9, 26.9, 52.4, 53.0, 105.5, 110.7, 111.4, 112.5, 118.0, 118.7, 119.5, 119.9, 121.4, 122.5, 122.8, 127.0, 128.9, 135.2, 135.5, 136.4, 169.9, 172.7. MS (ESI): *m/z* (%) = 390 $[M+H]^+$; 388 $[M-H]^-$. $C_{23}H_{23}N_3O_3$ (389.17): calcd. C 70.93, H 5.95, N 10.79; found C 70.81, H 6.03, N 10.89.

Methyl 2-acetamido-3-(2-benzyl-1-methyl-1H-indol-3-yl)propanoate (3i)

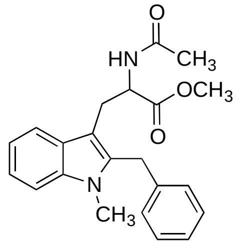

Yellow oil (186 mg, 51%). TLC: *Rf* = 0.25 (cyclohexane/ethyl acetate, 6:4; UV, p-anisaldehyde). FTIR (nujol): $\tilde{\nu}$max = 3281, 1744, 1659 cm^{-1}. ^{1}H NMR (200 MHz, $CDCl_3$): δ = 1.69 (s, 3H), 3,37 (d, *J*=5.5 Hz, 2H), 3.49 (s, 3H), 3.61 (s, 3H), 4.19 (s, 2H), 4.90 (ddd, J_1=J_2=5.5 Hz, J_3=7.5 Hz, 1H), 5.99 (br d, *J*=7.5 Hz, 1H), 7.04-7.08 (m, 2H), 7.10-7.22 (m, 2H), 7.25-7.35 (m, 4H), 7.52-7.56 (m, 1H). ^{13}C NMR (50 MHz, $CDCl_3$): δ = 22.9, 27.2, 30.1, 30.3, 52.4, 53.2, 106.9, 109.0, 118.34, 119.4, 121.5, 126.6, 127.8, 127.9, 128.9, 135.9, 137.0, 138.5, 169.8, 172.5. MS (ESI): *m/z* (%) = 365 $[M+H]^+$. $C_{21}H_{22}N_2O_3$ (364.18): calcd. C 72.50, H 6.64, N 7.69; found C 72.61, H 6.58, N 7.61.

Methyl 3-(2-benzyl-1*H*-indol-3-yl)-2-(1,3-dioxoisoindolin-2-yl)propanoate (3j)

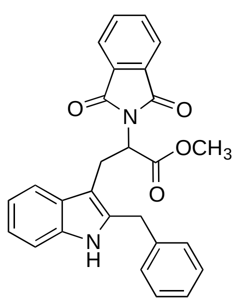

Compound **3j** was prepared according to the general procedure by using methyl 2-(1,3-dioxoisoindolin-2-yl)acrylate **2b** (1.2 equiv.) instead of **2a**.

Yellow solid (223 mg, 51%). mp: 125-126 °C (from ether-hexane); TLC: *Rf* = 0.21 (cyclohexane/ethyl acetate, 1:1; UV, p-anisaldehyde). FTIR (nujol): $\tilde{\nu}$max = 3394, 1774, 1716 cm^{-}

[1]. ^{1}H NMR (200 MHz, $CDCl_3$): δ = 3.79 (s, 3H), 3.74-3.83 (m, 2H), 4.01-4.18 (m, 2H), 5.28 (dd, J_1= 6.5 Hz, J_2=9.0 Hz, 1H), 6.95-7.22 (m, 8H), 7.53-7.73 (m, 6H) ppm. ^{13}C NMR (50 MHz, $CDCl_3$): δ = 24.2, 32.0, 52.5, 52.8, 107.5, 110.5, 118.1, 119.5, 121.5, 123.4, 126.6, 128.1, 128.6, 131.7, 133.9, 134.8, 135.4, 138.2, 167.5, 169.6 ppm. MS (ESI): *m/z* (%) = 439 $[M+H]^+$; 437 $[M-H]^-$. $C_{27}H_{22}N_2O_4$ (438.16): calcd. C 73.96, H 5.06, N 6.39; found C 74.11, H 5,11 N 6.33.

Methyl 2-acetamido-3-(2-allyl-1*H*-indol-3-yl)propanoate (3k)

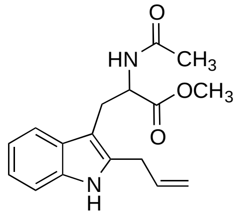

Brown solid (183 mg, 61%). mp: 100-101 °C (from ether-hexane); TLC: *Rf* = 0.31 (cyclohexane/ethyl acetate, 1:1; UV, p-anisaldehyde). FTIR (nujol): $\tilde{\nu}$max = 3403, 3303, 1737, 1662 cm^{-1}. ^{1}H NMR (200 MHz, $CDCl_3$): δ = 1.94 (s, 3H), 3.29 (d, J=5.5 Hz, 2H), 3.45-3.49 (m, 2H), 3.68 (s, 3H), 4.91 (ddd, J_1=J_2=5.5 Hz, J_3=8.0 Hz, 1H), 5.16-5.20 (m, 1H), 5.24-5.26 (m, 1H), 5.85-6.05 (m, 2H), 7.05-7.19 (m, 2H), 7.29 (dd, J_1=2.0 Hz, J_2=6.0 Hz, 1H), 7.46 (dd, J_1=2.0 Hz, J_2=6.0 Hz, 1H), 8.00 (br s, 1H). ^{13}C NMR (50 MHz, $CDCl_3$): δ = 23.3, 26.7, 30.5, 52.4, 53.0, 106.0, 110.6, 117.7, 118.1, 119.6, 121.6, 128.9, 134.0, 134.9, 135.4, 169.7, 172.5. MS (ESI): *m/z* (%) = 301 $[M+H]^+$; 299 $[M-H]^-$. $C_{17}H_{20}N_2O_3$ (300.15): calcd. C 67.98, H 6.71, N 9.33; found C 68.07, H 6.78, N 9.41.

Methyl 2-acetamido-3-(2-(3-methylbut-2-enyl)-1*H*-indol-3-yl)propanoate (3l)

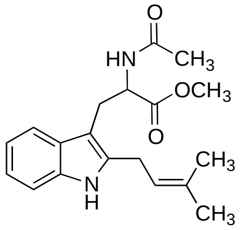

Yellow solid (282 mg, 86%). mp: 87-88 °C (from ether-hexane); TLC: *Rf* = 0.33 (cyclohexane/ethyl acetate, 1:1; UV, p-anisaldehyde). FTIR (nujol): $\tilde{\nu}$max = 3403, 3315, 1736, 1659 cm^{-1}. ^{1}H NMR (200 MHz, $CDCl_3$): δ = 1.77 (s, 3H), 1.79 (s, 3H), 1.92 (s, 3H), 3.28 (dd, J_1=2.0 Hz, J_2=5.5 Hz, 2H), 3.41 (d, J=7.0 Hz, 2H), 3.68 (s, 3H), 4.91 (ddd, J_1=J_2=5.5 Hz, J_3=7.5 Hz, 1H), 5.25-5.32 (m, 1H), 6.12 (d, J=7.5 Hz, 1H), 7.03-7.16 (m, 2H), 7.25-7.29 (m, 1H), 7.42-7.47 (m, 1H), 8.20 (br s, 1H). ^{13}C NMR (50 MHz, $CDCl_3$): δ = 17.9, 23.1, 25.0, 25.8, 26.7, 52.4, 53.0, 104.9, 110.6, 117.9, 119.5, 120.2, 121.3, 129.0, 135.0, 135.3, 136.2, 169.9, 172.6. MS (ESI): *m/z* (%) = 329 $[M+H]^+$; 327 $[M-H]^-$. $C_{19}H_{24}N_2O_3$ (328.18): calcd. C 69.49, H 7.37, N 8.53; found C 69.62, H 7.31, N 8.62.

Methyl 2-acetamido-3-(2-((E)-3,7-dimethylocta-2,6-dienyl)-1*H*-indol-3-yl)propanoate (3m)

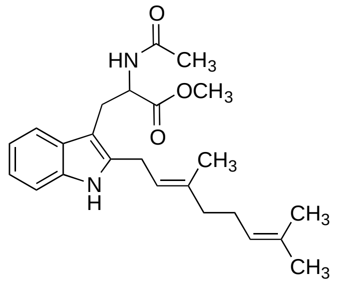

Yellow solid (277 mg, 70%). mp: 84-85 °C (from ether-hexane); TLC: *Rf* = 0.42 (cyclohexane/ethyl acetate, 1:1; UV, p-anisaldehyde). FTIR (nujol): $\tilde{\nu}$max = 3471, 3297, 1736, 1658 cm^{-1}. ^{1}H NMR (200 MHz, $CDCl_3$): δ = 1.65 (s, 3H), 1.75 (s, 6H), 1.95 (s, 3H), 2.09-2.18 (m, 4H) 3.30 (d, J=5.5 Hz, 2H), 3.44 (d, J=7.0 Hz, 2H), 3.70 (s, 3H), 4.92 (ddd, J_1=J_2=5.5 Hz, J_3=7.5 Hz, 1H), 5.10-5.13 (m, 1H), 5.27-5.34 (m, 1H), 6.07 (d, J=7.5 Hz, 1H), 7.05-7.17 (m, 2H), 7.26-7.30 (m, 1H), 7.43-7.47 (m, 1H), 7.97 (br s, 1H). ^{13}C NMR (50 MHz, $CDCl_3$): δ = 16.2, 17.8, 23.2, 24.8, 25.8, 26.3, 26.8, 39.6, 52.4, 52.9, 104.9, 110.5, 117.9, 119.5, 119.9, 121.3, 123.9, 129.1, 132.0, 135.1, 136.0, 138.9, 169.6, 172.6. MS (ESI): *m/z* (%) = 397 $[M+H]^+$; 395 $[M-H]^-$. $C_{24}H_{32}N_2O_3$ (396.24): calcd. C 72.70, H 8.13, N 7.06; found C 72.55, H 8.05, N 7.00.

Methyl 2-acetamido-3-(2-(methylthio)-1*H*-indol-3-yl)propanoate (3o)

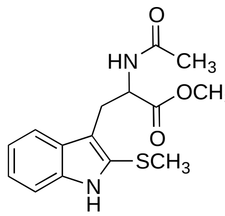

Brown solid (208 mg, 68%). mp: 118-119 °C (from ether-hexane); TLC: *Rf* = 0.25 (cyclohexane/ethyl acetate, 1:1; UV, p-anisaldehyde). FTIR (nujol): $\tilde{\nu}$max = 3379, 3277, 1748, 1659 cm^{-1}. ^{1}H NMR (200 MHz, $CDCl_3$): δ = 1.96 (s, 3H), 2.38 (s, 3H), 3.38 (dd, J_1=4.5 Hz, J_2=5.5 Hz, 2H), 3.70 (s, 3H), 4.93 (ddd, J_1=J_2=5.5 Hz, J_3=8.0 Hz, 1H), 6.24 (d, J=8.0 Hz, 1H), 7.06-7.25 (m, 3H), 7.50 (d, J=7.5 Hz, 1H), 8.77 (br s, 1H). ^{13}C NMR (50 MHz, $CDCl_3$): δ = 19.7, 23.2, 27.3, 52.5, 53.0, 110.8, 114.1, 118.6, 120.0, 123.0, 128.0, 128.4, 136.6, 169.9, 172,4. MS (ESI): *m/z* (%) = 307 $[M+H]^+$; 305 $[M-H]^-$. $C_{15}H_{18}N_2O_3S$ (306.10): calcd. C 58.80, H 5.92, N 9.14; found C 58.96, H 5.88, N 9.05.

Methyl 1-acetyl-3a-benzyl-1,2,3,3a,8,8a-hexahydropyrrolo[2,3-*b*]indole-2-carboxylate (4a)

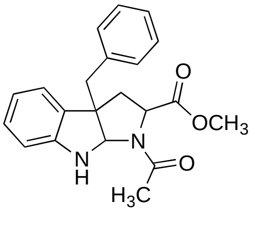

Compound **4a** was prepared according to the general procedure by quenching the reaction after 5 hours. The separation of diastereoisomers was performed on preparative thin layer chromatography (silica gel 60 F254 0.5 mm). The ratio of the diastereoisomers of the *exo*-*endo* compounds is 3:1, it

was determined by HPLC [Merck Purospher-Star™ RP 18 column, End-capped (250x4x5 micron) and methanol/water, with 5% of formic acid, as eluent (gradient from 40% to 90% of methanol in 15 minutes, flow of 0.8 mL/min)].

(±)-endo-Methyl 1-acetyl-3a-benzyl-1,2,3,3a,8,8a-hexahydropyrrolo[2,3-*b*]indole-2-carboxylate

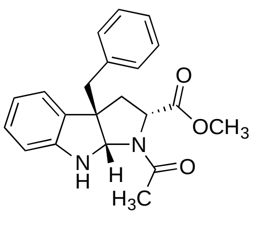

Brown solid (25 mg, 7%). mp: 236-238 °C (from ether-hexane); TLC: *Rf* = 0.26 (cyclohexane/ethyl acetate, 1:1; UV, p-anisaldehyde). HPLC: t_r 13.15 min. FTIR (nujol): $\tilde{\nu}$max = 3393, 1736, 1647 cm^{-1}. ^{1}H NMR (200 MHz, $CDCl_3$): δ = 1.87 (s, 3H), 2.50 (dd, J_1=8.0 Hz, J_2=13.0 Hz, 1H), 2.65 (d, *J*=13.0 Hz, 1H), 2.83 (d, *J*=13.5 Hz, 1H), 2.91 (d, *J*=13.5 Hz, 1H), 3.07 (s, 3H), 4.28 (d, *J*=8.0 Hz, 1H), 5.17 (br s, 1H), 5.29 (s, 1H), 6.48-6.62 (m, 3H), 6.93-7.02 (m, 3H), 7.17-7.24 (m, 3H). ^{13}C NMR (50 MHz, $CDCl_3$): δ = 22.1, 29.7, 38.6, 52.3, 55.8, 60.7, 80.5, 109.4, 118.3, 124.4, 126.8, 128.1, 128.9, 129.9, 130.4, 136.7, 150.1, 171.0, 171.0. MS (ESI): *m/z* (%) = 351 $[M+H]^+$. $C_{21}H_{22}N_2O_3$ (350.16): calcd. C 71.98, H 6.33, N 7.99; found C 71.89, H 6.41, N 7.91.

(±)-exo-Methyl 1-acetyl-3a-benzyl-1,2,3,3a,8,8a-hexahydropyrrolo[2,3-*b*]indole-2-carboxylate

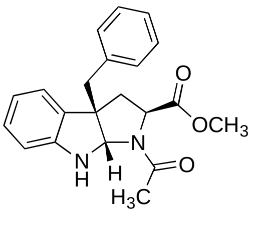

Brown solid (63 mg, 18%). mp: 182-184 °C (from ether-hexane); TLC: *Rf* = 0.34 (cyclohexane/ethyl acetate, 1:1; UV, p-anisaldehyde). HPLC: t_r 13.67 min. FTIR (nujol): $\tilde{\nu}$max = 3399, 1742, 1642 cm^{-1}. ^{1}H and ^{13}C NMR spectra of this compound showed the presence of two rotamers. ^{1}H and ^{13}C NMR signals corresponding to major rotamer were indicated with * while the signals corresponding to minor rotamer were indicated with §. ^{1}H NMR (200 MHz, $CDCl_3$): δ = 1.75 (s, **3H***), 2.09 (s, *1.4H*§), 2.26 (dd, J_1=9.0 Hz, J_2=13 Hz, *0.47H*§), 2.33 (dd, J_1=8.0 Hz, J_2=12.5 Hz, **1H***), 2.49 (dd, J_1=7.0 Hz, J_2=12.5 Hz, *0.47H*§), 2.61 (dd, J_1=8.0 Hz, J_2=13.0 Hz, **1H***), 2.79 (d, *J*=13.5 Hz, **1H***), 2.94 (d, *J*=13.5 Hz, **1H***), 2.95 (d, *J*=13.0 Hz, *0.47H*§), 3.07 (d, *J*=13.0 Hz, *0.47H*§), 3.68 (s, **3H***), 3.68 (s, *1.4H*§) 4.07 (dd, J_1=7.0 Hz, J_2=9.0 Hz, *0.47H*§), 4.09 (dd, J_1=J_2=8.0 Hz, **1H***), 4.22 (br s, *0.47H*§), 5.33 (s, *0.47H*§), 5.38 (s, **1H***), 5.47 (br s, **1H***), 6.47-7.55 (m, **9H***), 6.47-7.55 (m, *4.2H*§). ^{13}C NMR (50 MHz, $CDCl_3$): δ = 22.4§, 22.6*, 39.6§, 40.3*,42.7*, 44.0§, 52.3§,52.8*, 55.7*, 59.2§, 59.4§, 60.0*, 80.8§, 82.1*, 109.8*, 110.9§, 118.5*, 120.3§, 123.7§, 123.8*, 126.8*, 126.8§, 128.1*, 128.1§, 128.7*, 128.9§, 130.0*, 130.3*, 130.3§, 131.7§, 136.6*, 136.7§, 148.1§, 148.6*, 169.8§, 171.3*, 172.6§, 173.0*. MS (ESI): *m/z* (%) = 351 $[M+H]^+$. $C_{21}H_{22}N_2O_3$ (350.16): calcd. C 71.98, H 6.33, N 7.99; found C 72.14, H 6.38, N 8.06.

Resubmission of Methyl 1-acetyl-3a-benzyl-1,2,3,3a,8,8a-hexahydropyrrolo[2,3-*b*]indole-2-carboxylate (4a) to the general procedure.

Compound **4a** was resubmitted to the general reaction conditions and after 16 h gave **3a** in 89% yield.

COSY and NOESY Spectra of 3a

^{1}H COSY of 3a

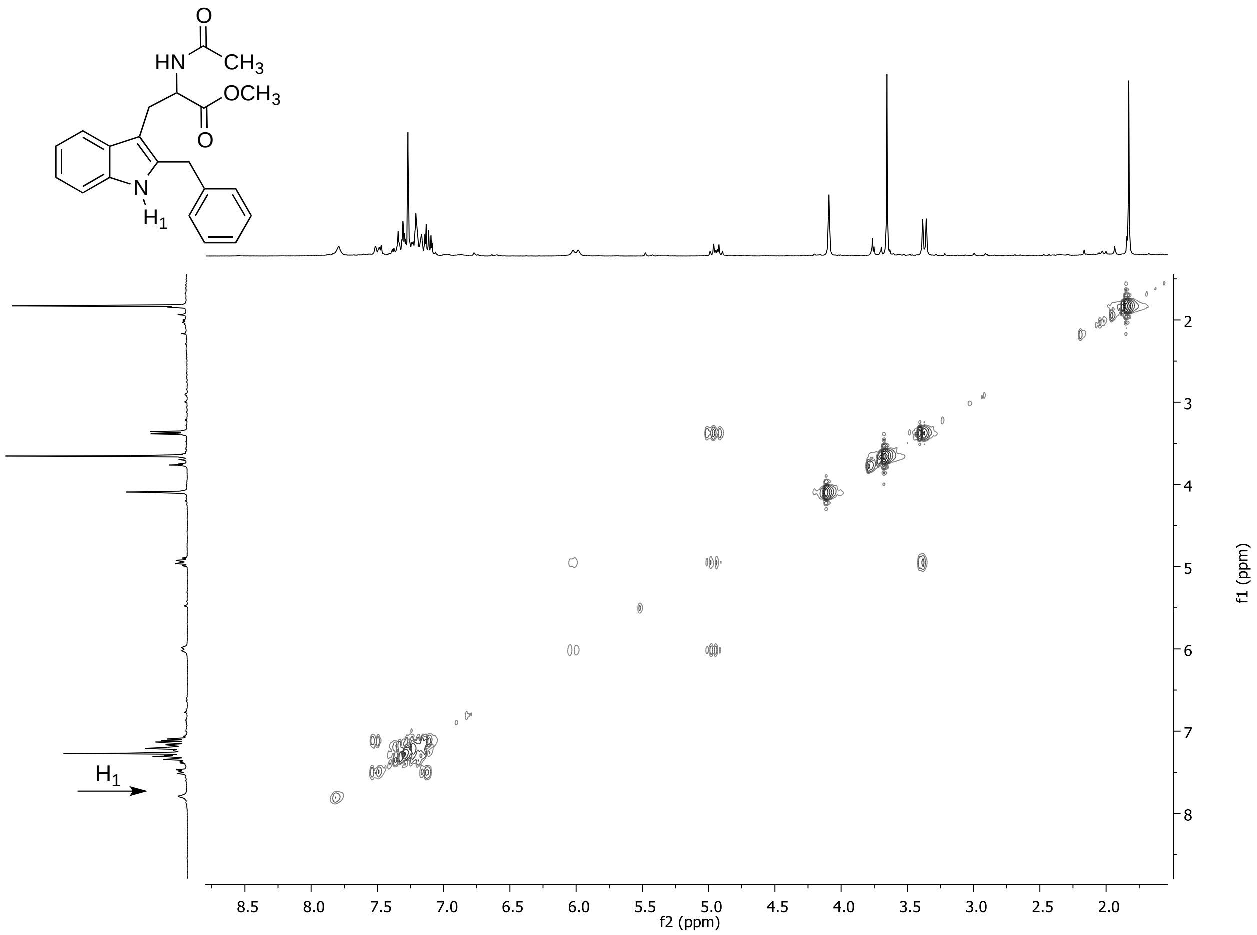

No correlation between H-1 (NH) and the other hydrogens of the molecule (no cross peaks), so there are no Hydrogens linked to C-2, that carbon have a substituent.

^{1}H NOESY of 3a

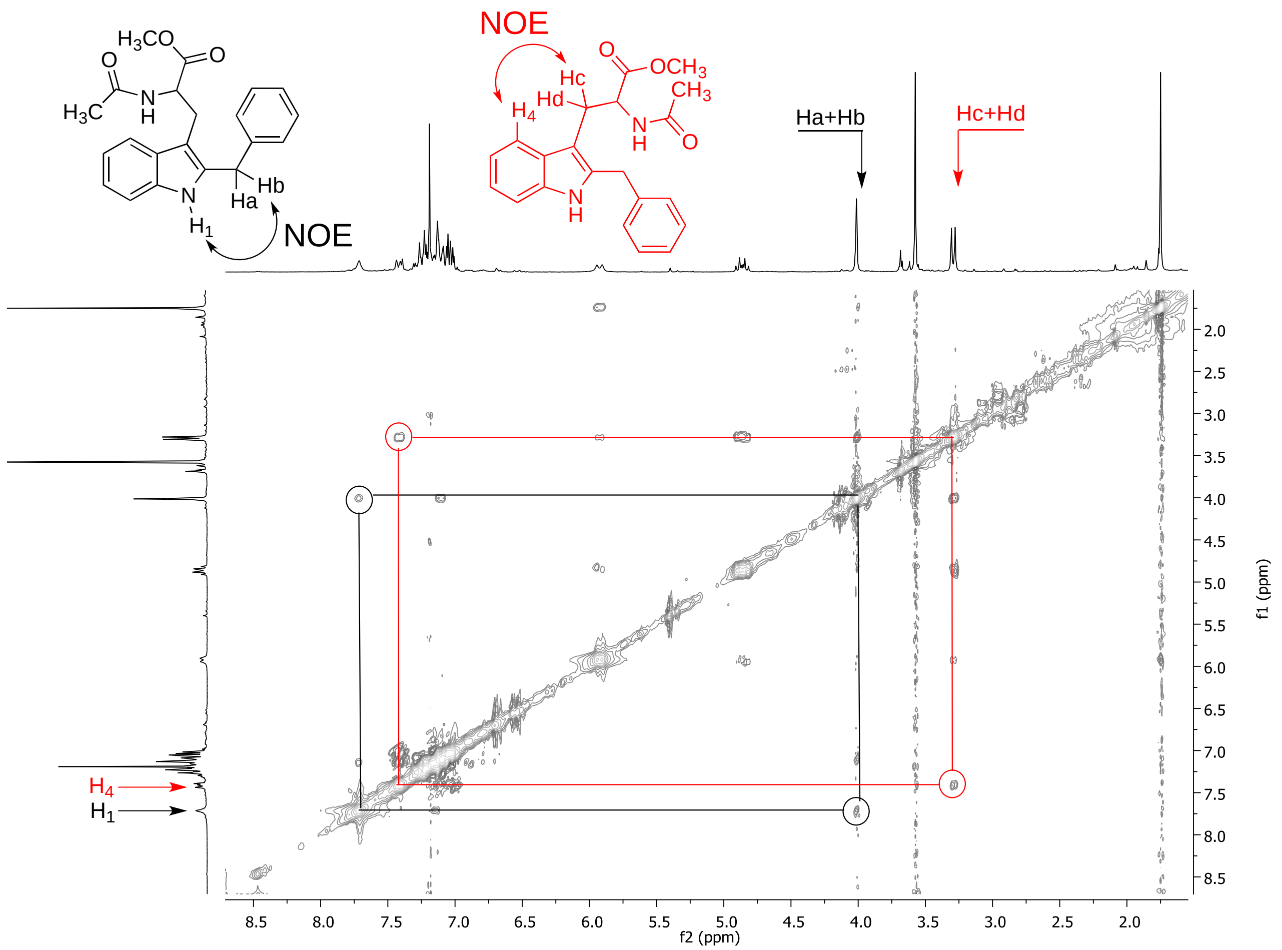

Nuclear Overhauser effect between H-1 and Ha+Hb, H-4 and Hc+Hd, the cross peaks confirm that those Hydrogens are spatially close demonstrating that the compound was 2-benzyl-N-acetyltryptophan methyl ester.

References

1. Angelovsky, G.; Keränen, M. D.; Linnepe, P.; Grudzielanek, S.; Eilbracht, P. *Adv. Synth. Catal.* **2006**, *348*, 1193-1199.
2. Whitney, S.; Grigg, R.; Derrick, A.; Keep, A. *Org. Lett*. **2007**, *9*, 3299-3302.
3. De Rosa, M.; Soriente, A. *Eur. J. Org. Chem*. **2010**, 1029-1032.
4. Yasuda, M.; Somyo, T.; Baba, A. *Angew. Chem. Int. Ed*. **2006**, *45*, 793-796.
5. Kimura M.; Futamada M.; Mukai R.; Tamaru Y. *J. Am. Chem. Soc.* **2005**, *127*, 4592-4593.
6. Westermaier, M.; Mayr, H. *Org. Lett.* **2006**, *8*, 4791-4794.
7. Fang, X.-L.; Tang, R.-Y.; Zhong, P.; Li, J.-H. *Sytnhesis* **2009**, *24*, 4183-4189.
8. Trost, B. M.; Dake, G. R. *J. Am. Chem. Soc*. **1997**, *119*, 7595-7596.
9. Yadav, J. S.; Subba Reddy, B. V.; Aravind, S.; Narayana Kumar, G. G. K. S.; Srinivas Reddy, A. *Tetrahedron Letters*. **2007**, *48*, 6117-6120.

^{1}H and ^{13}C NMR Spectra

^{1}H and ^{13}C NMR Spectrum of 3-(1,2,3,4-Tetrahydronaphthalen-1-yl)-1*H*-indole (1f)

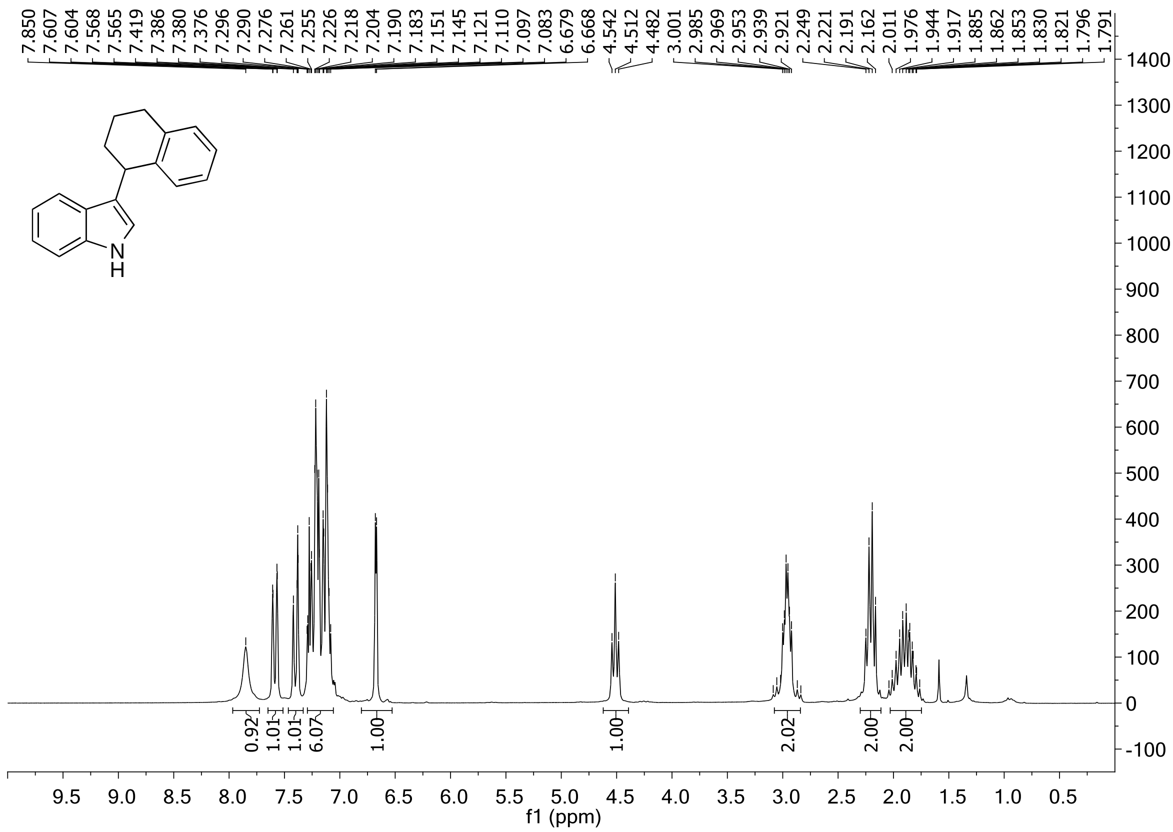

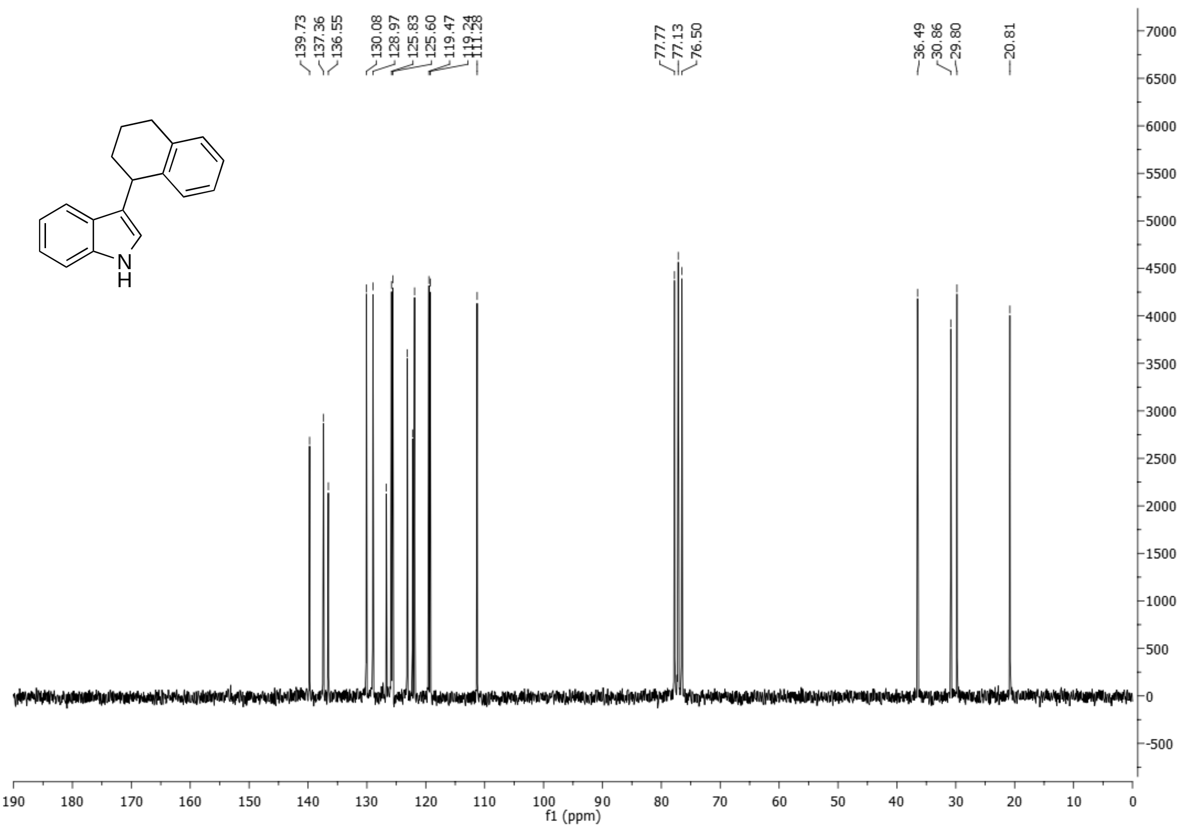

^{1}H and ^{13}C NMR Spectrum of Methyl 2-acetamido-3-(2-benzyl-1*H*-indol-3-yl)propanoate (3a)

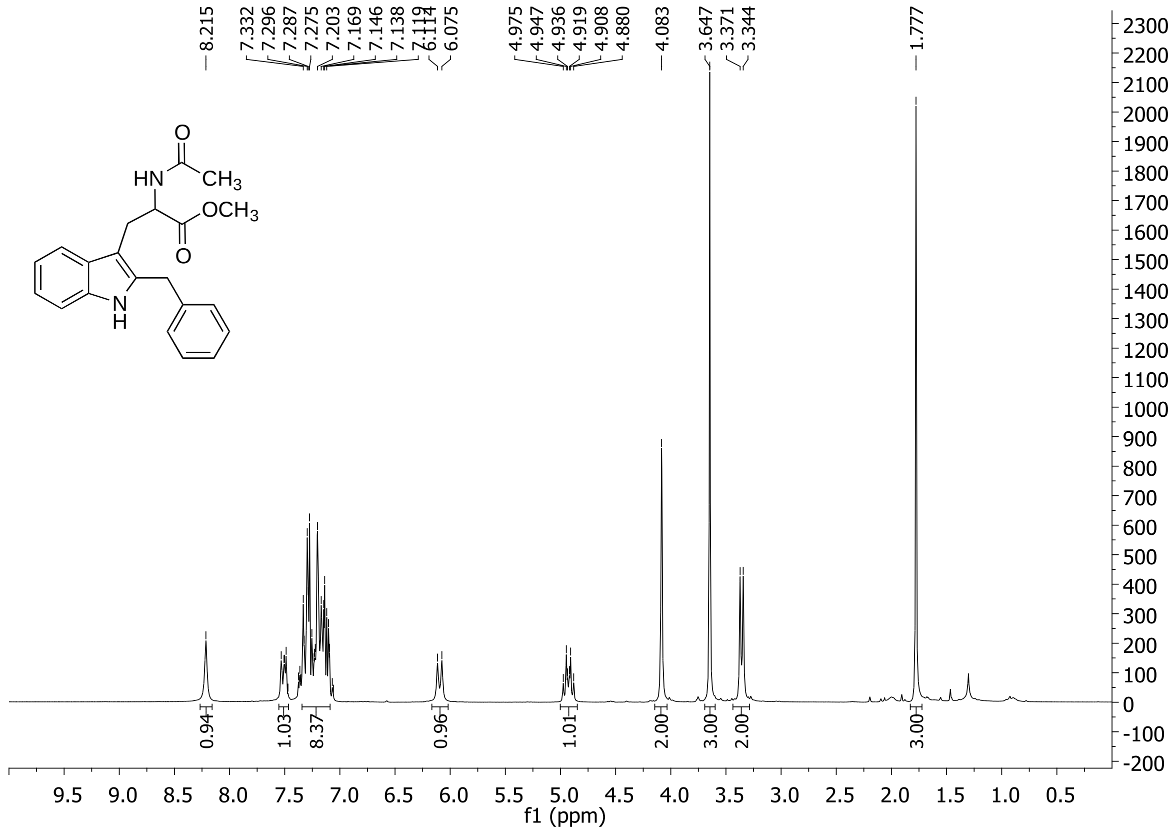

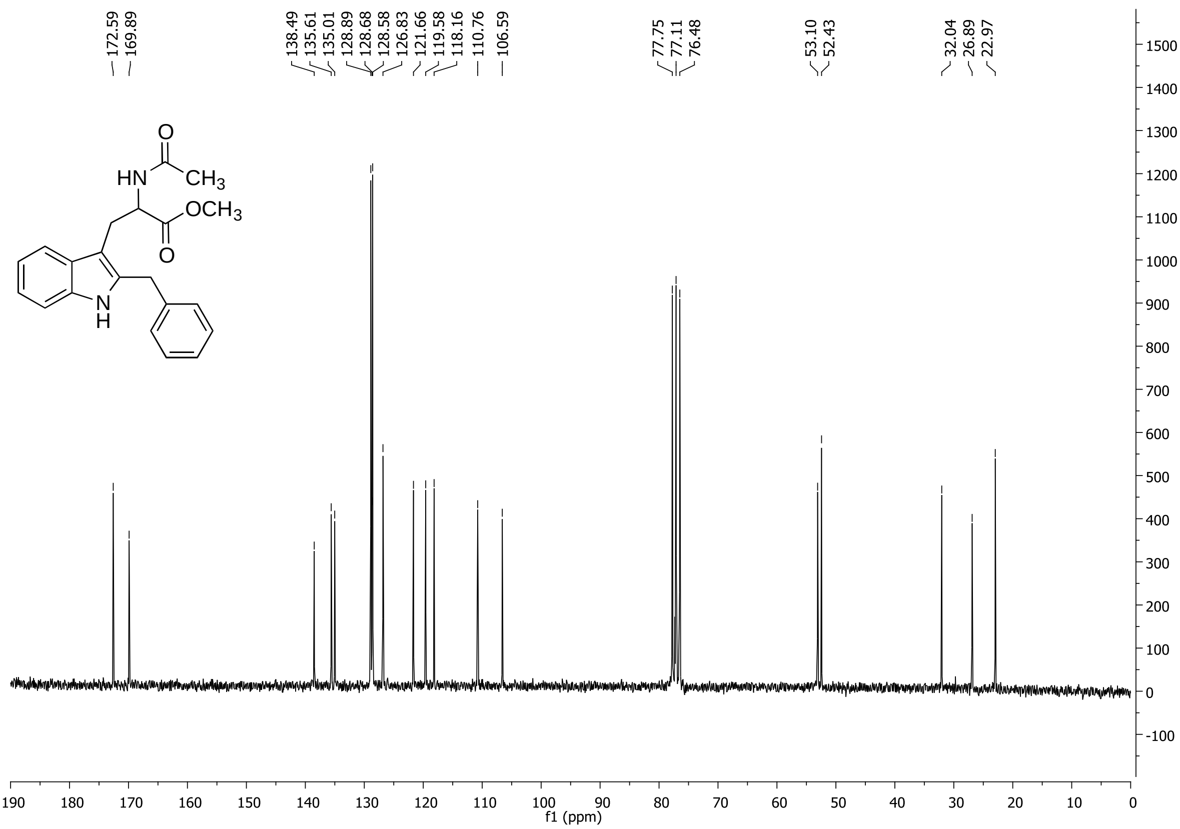

^{1}H and ^{13}C NMR Spectrum of Methyl 3-(2-(4-methoxybenzyl)-1*H*-indol-3-yl)-2-acetamidopropanoate (3b)

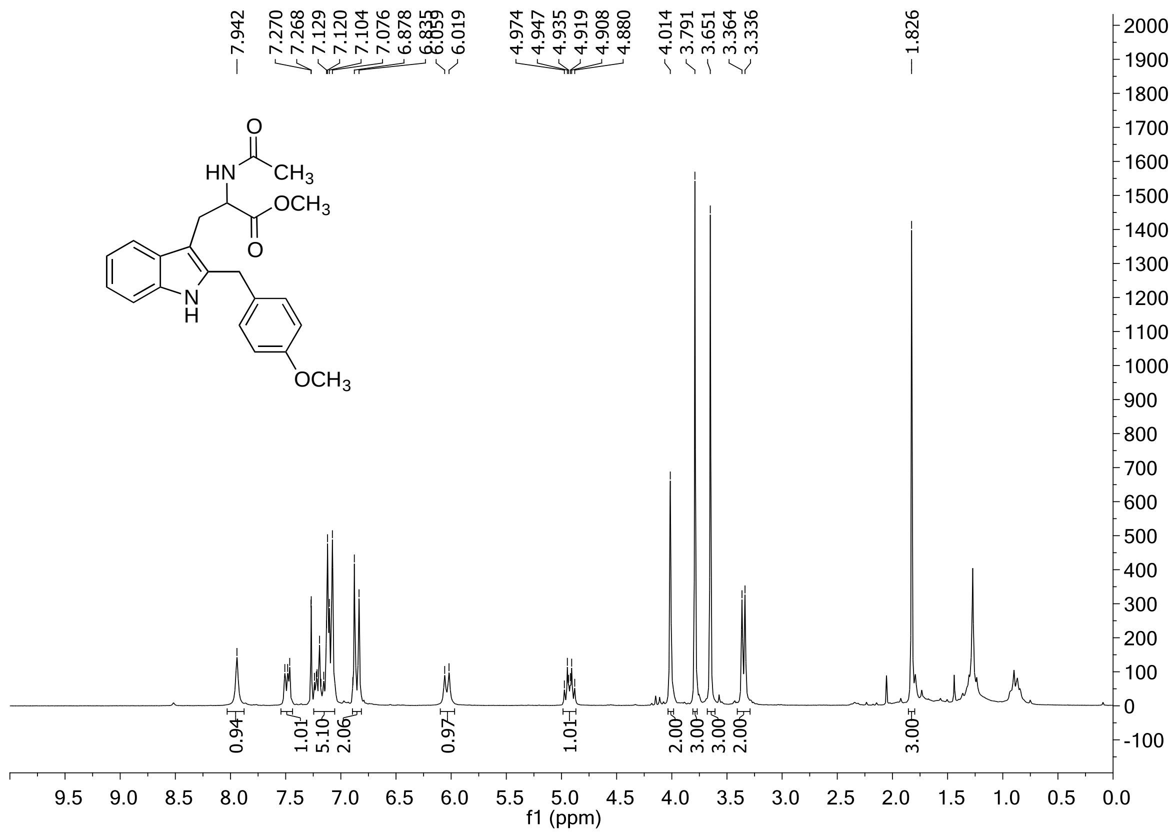

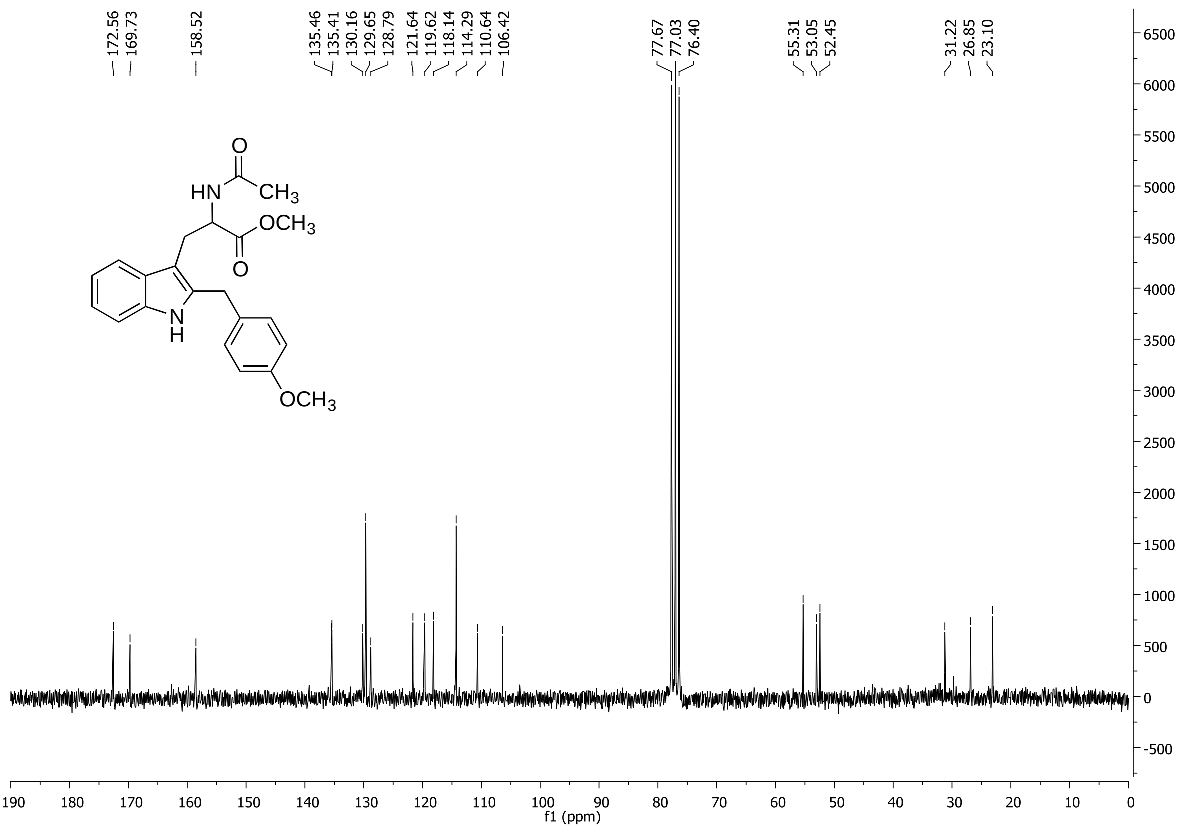

^{1}H and ^{13}C NMR Spectrum of Methyl 3-(2-(4-chlorobenzyl)-1*H*-indol-3-yl)-2-acetamidopropanoate (3c)

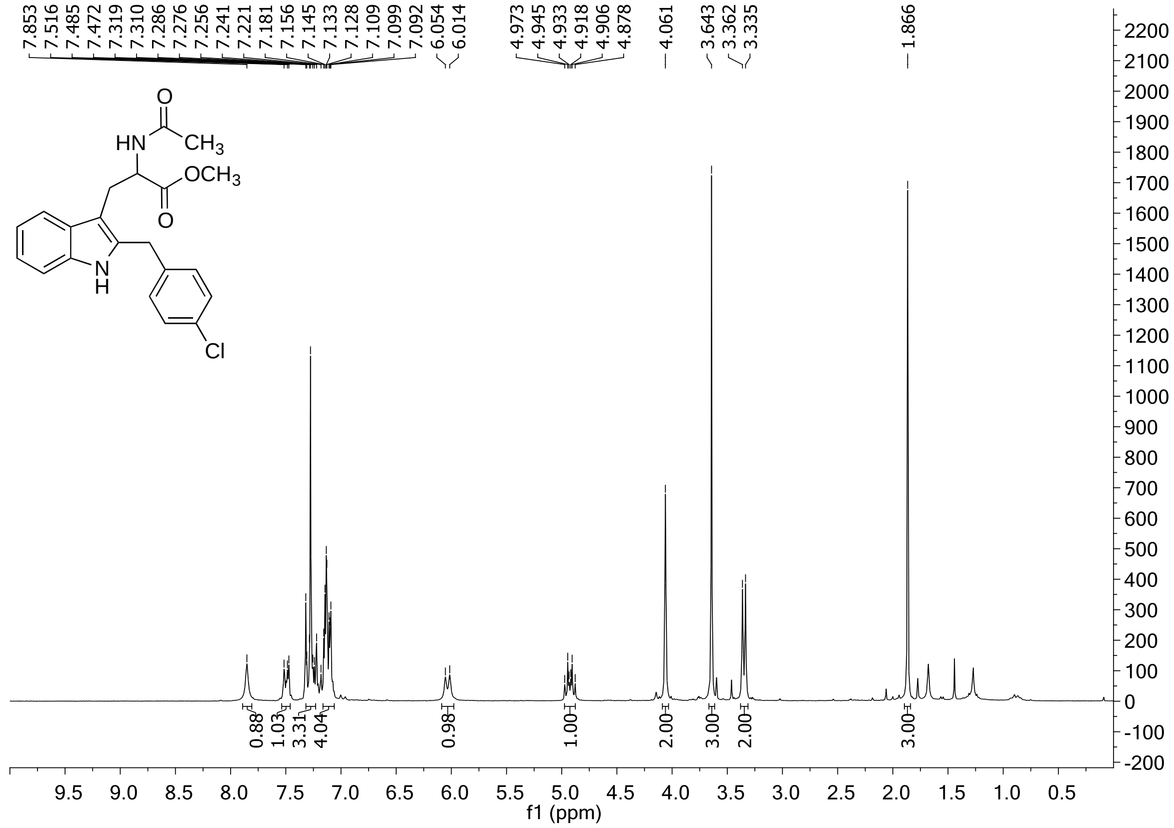

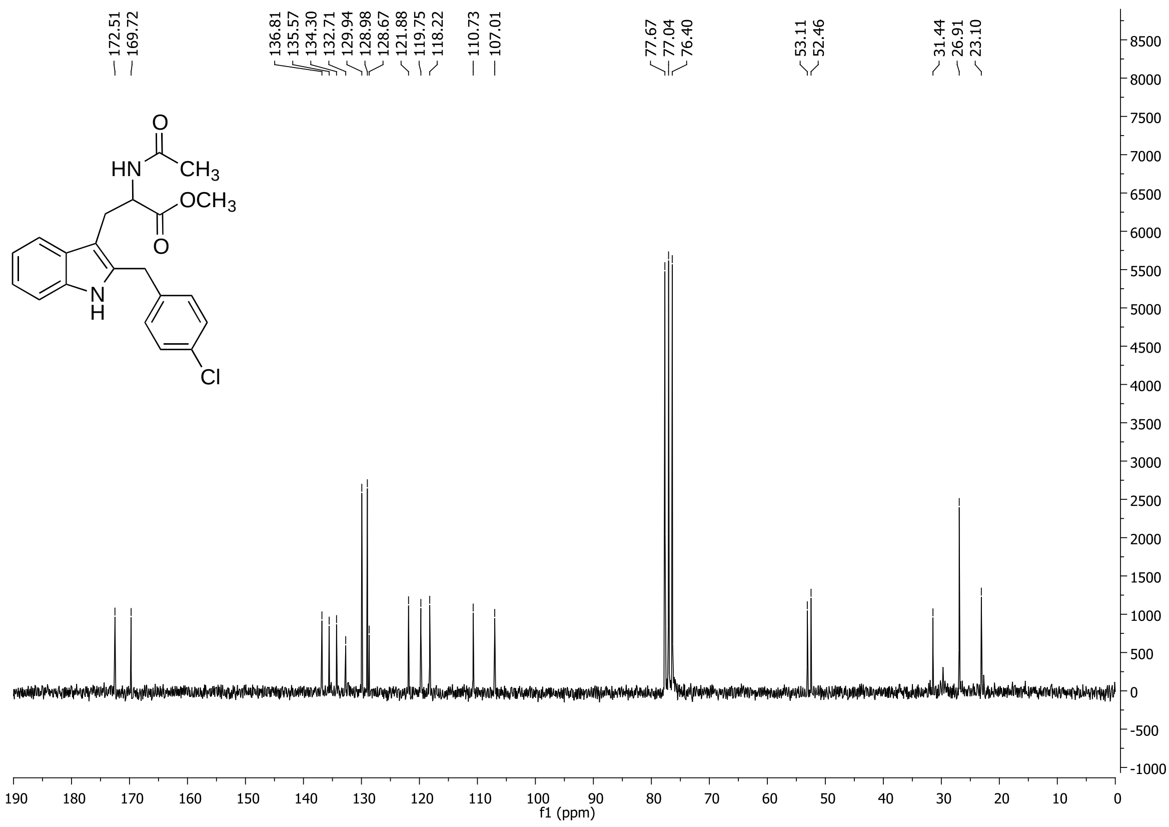

^{1}H and ^{13}C NMR Spectrum of Methyl 2-acetamido-3-(2-(1,2,3,4-tetrahydronaphthalen-1-yl)-1*H*-indol-3-yl)propanoate (3f)

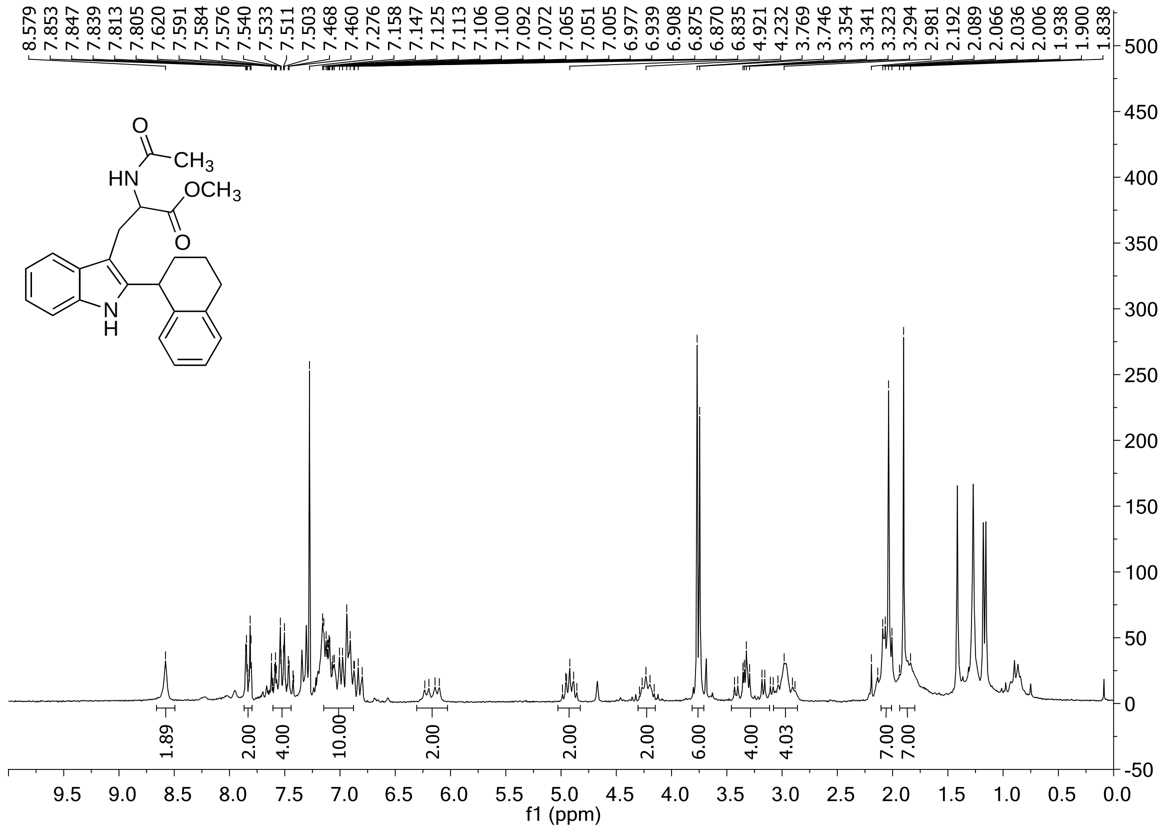

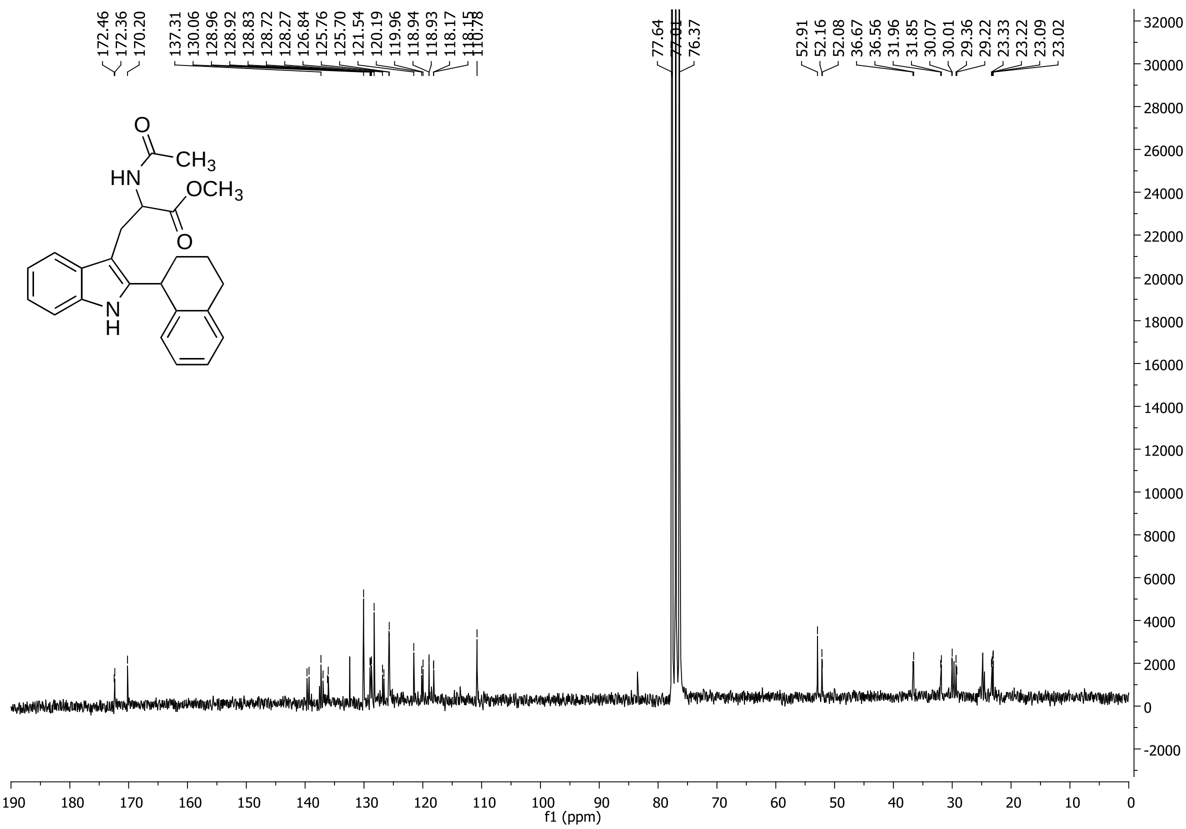

^{1}H and ^{13}C NMR Spectrum of Methyl 3-(2-((1*H*-indol-3-yl)methyl)-1*H*-indol-3-yl)-2-acetamidopropanoate (3h)

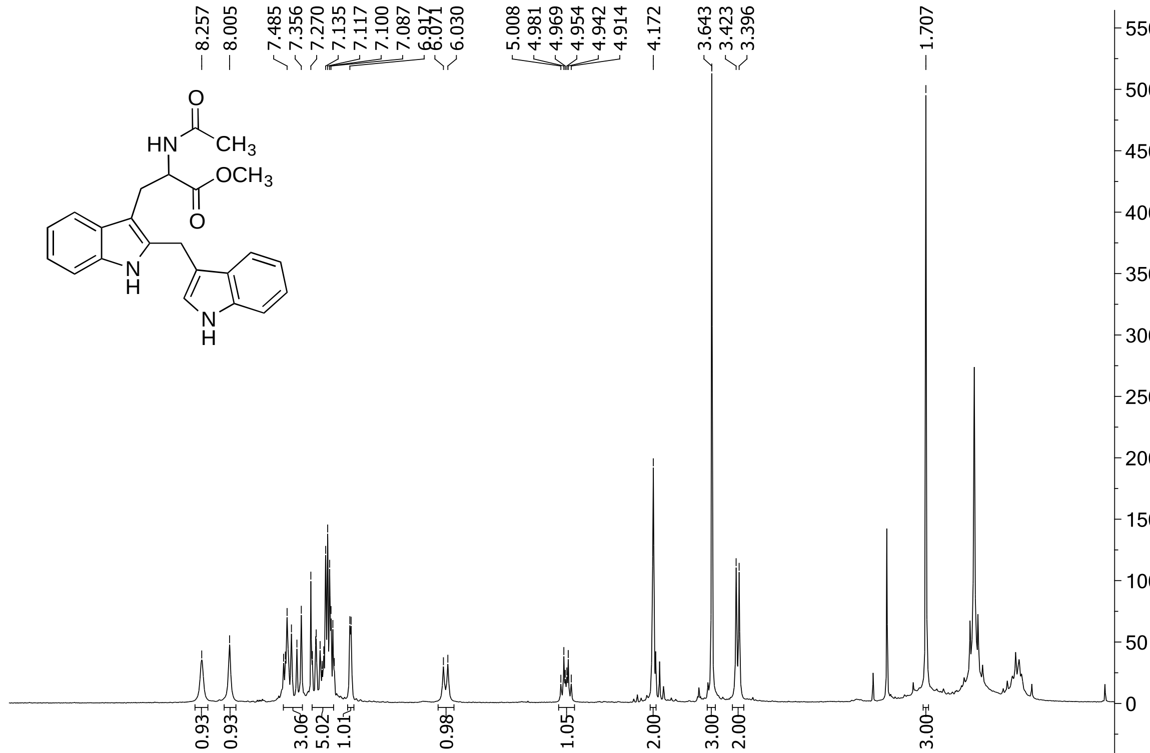

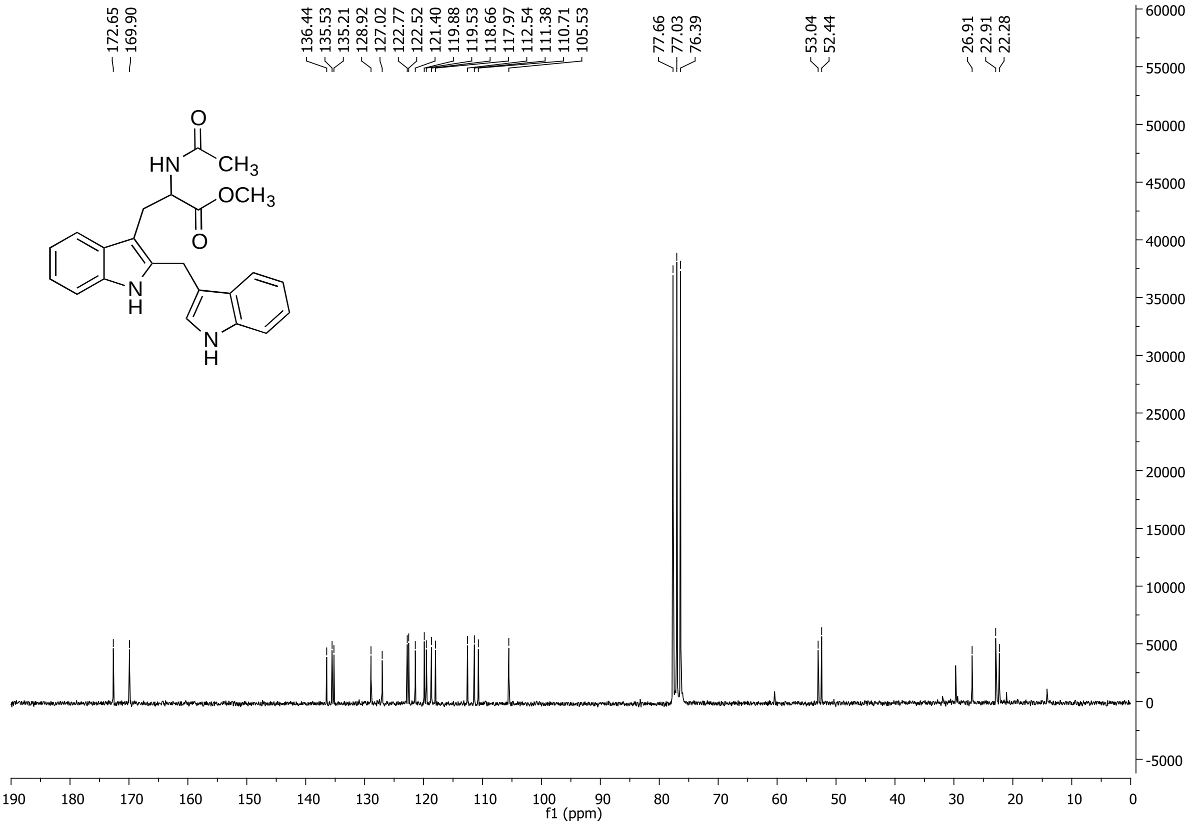

^{1}H and ^{13}C NMR Spectrum of Methyl 2-acetamido-3-(2-benzyl-1-methyl-1H-indol-3-yl)propanoate (3i)

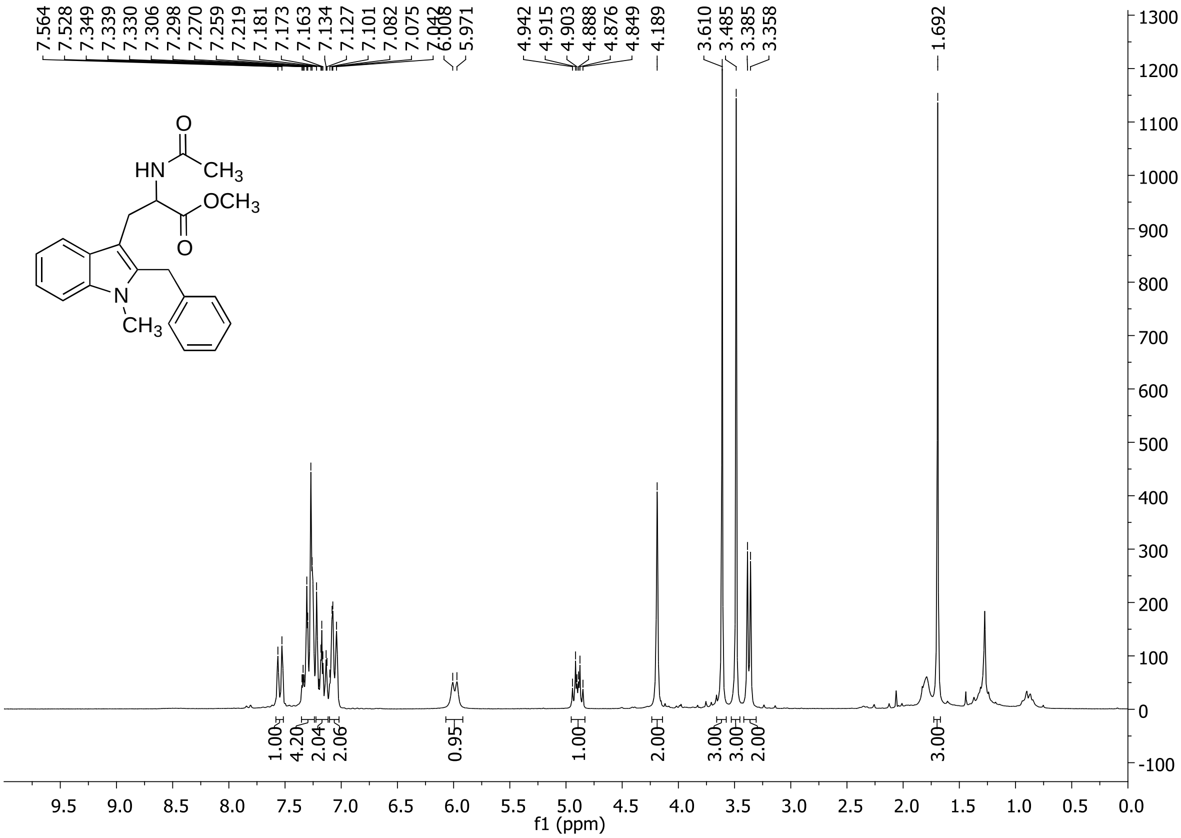

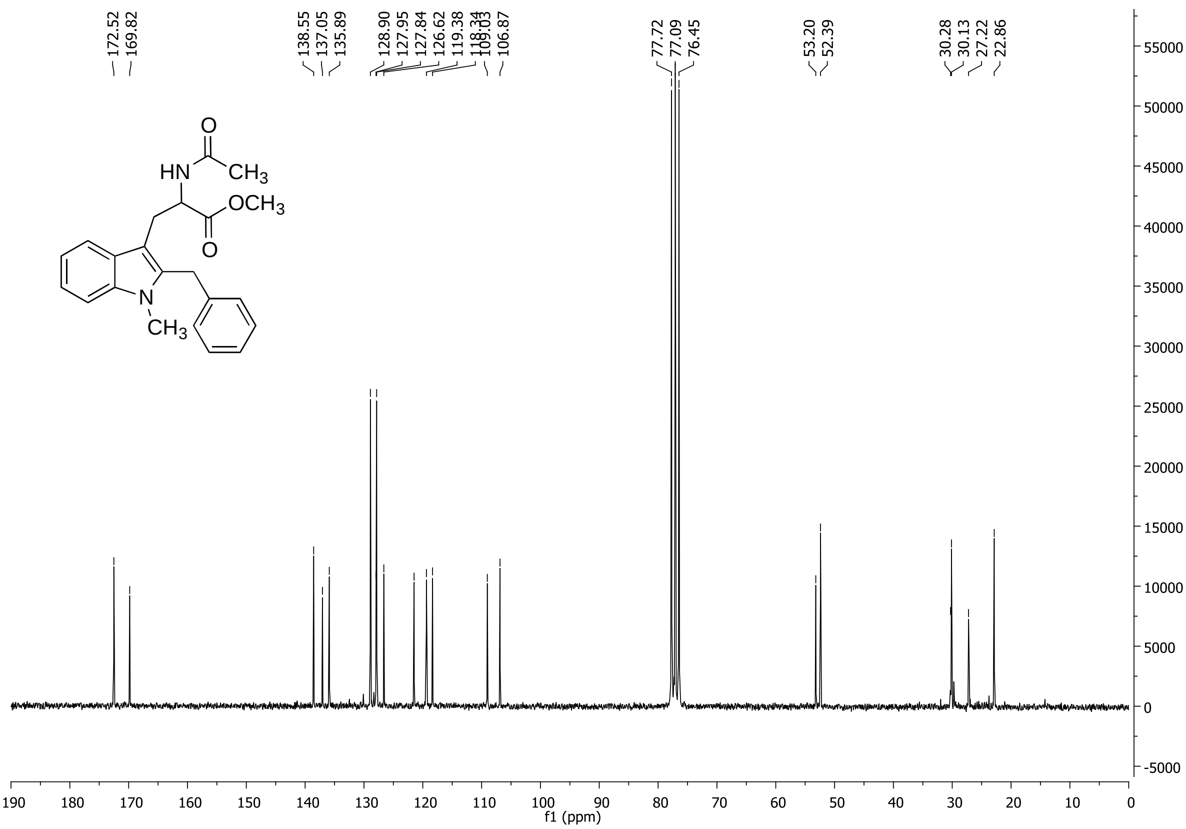

^{1}H and ^{13}C NMR Spectrum of methyl 3-(2-benzyl-1H-indol-3-yl)-2-(1,3-dioxoisoindolin-2-yl)propanoate (3j)

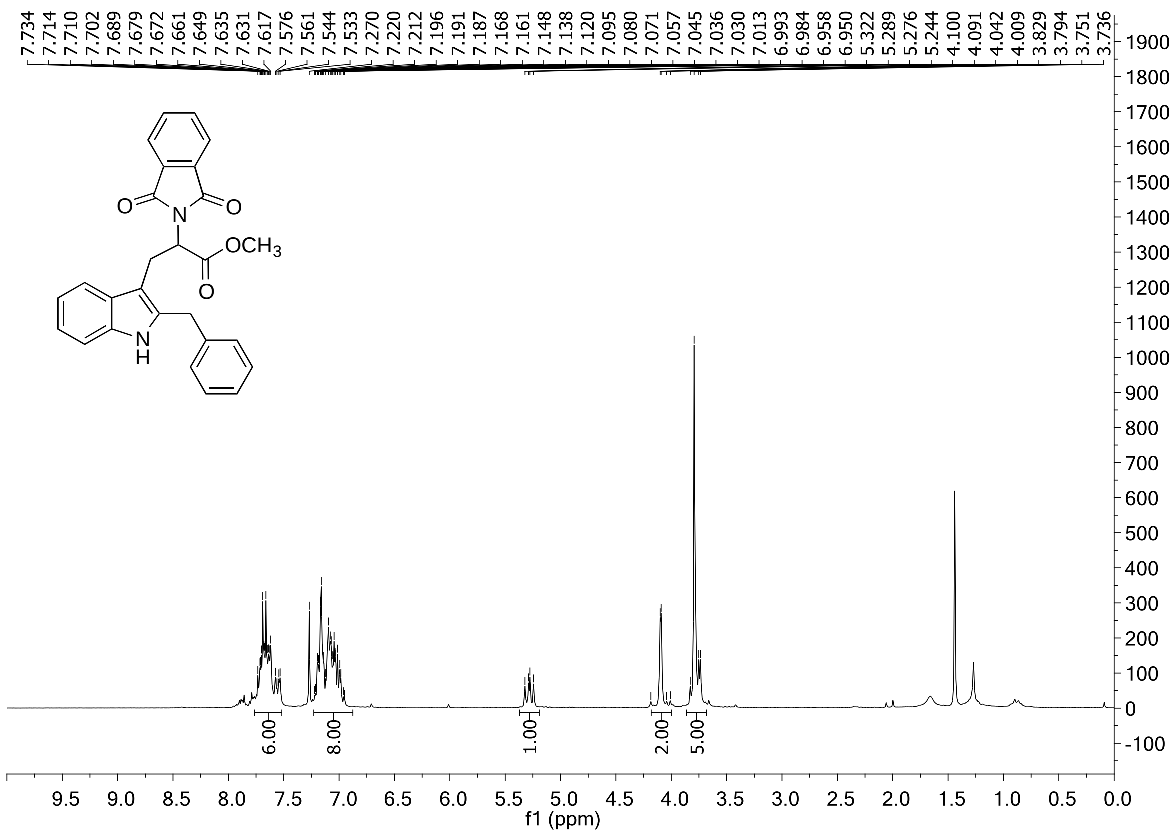

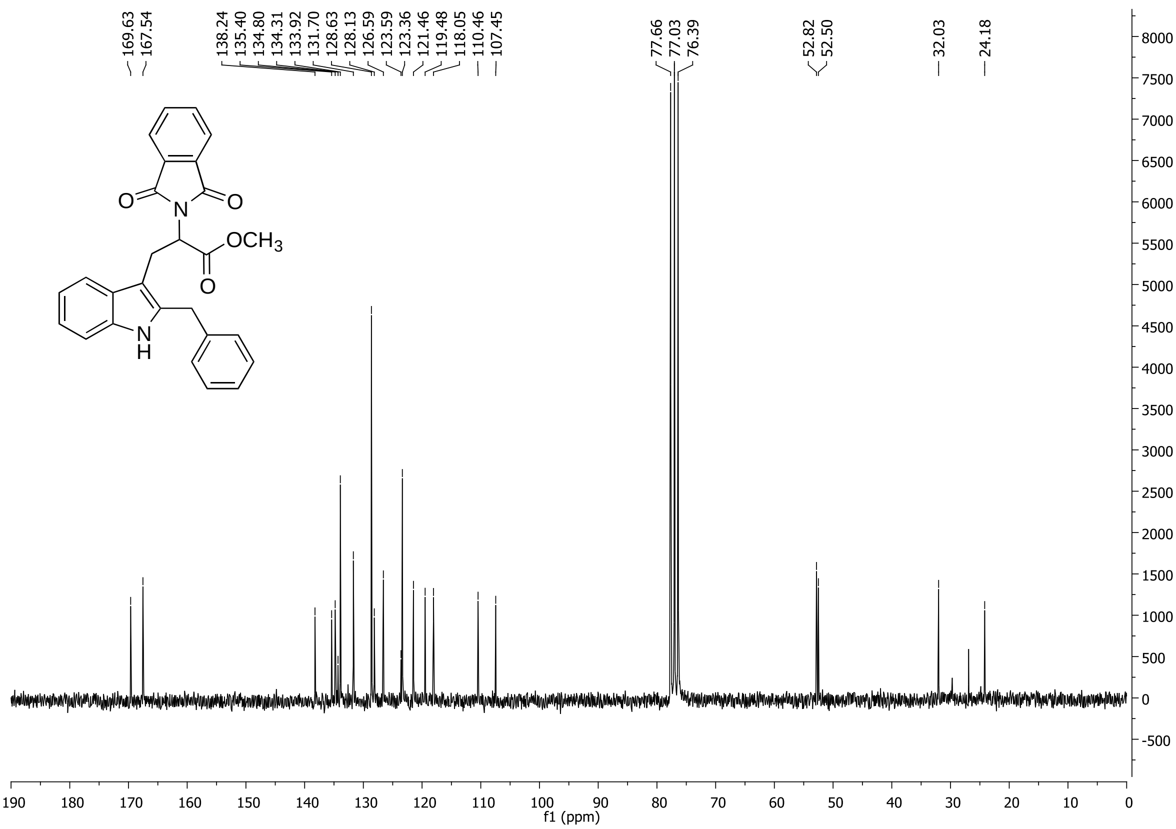

^{1}H and ^{13}C NMR Spectrum of Methyl 2-acetamido-3-(2-allyl-1*H*-indol-3-yl)propanoate (3k)

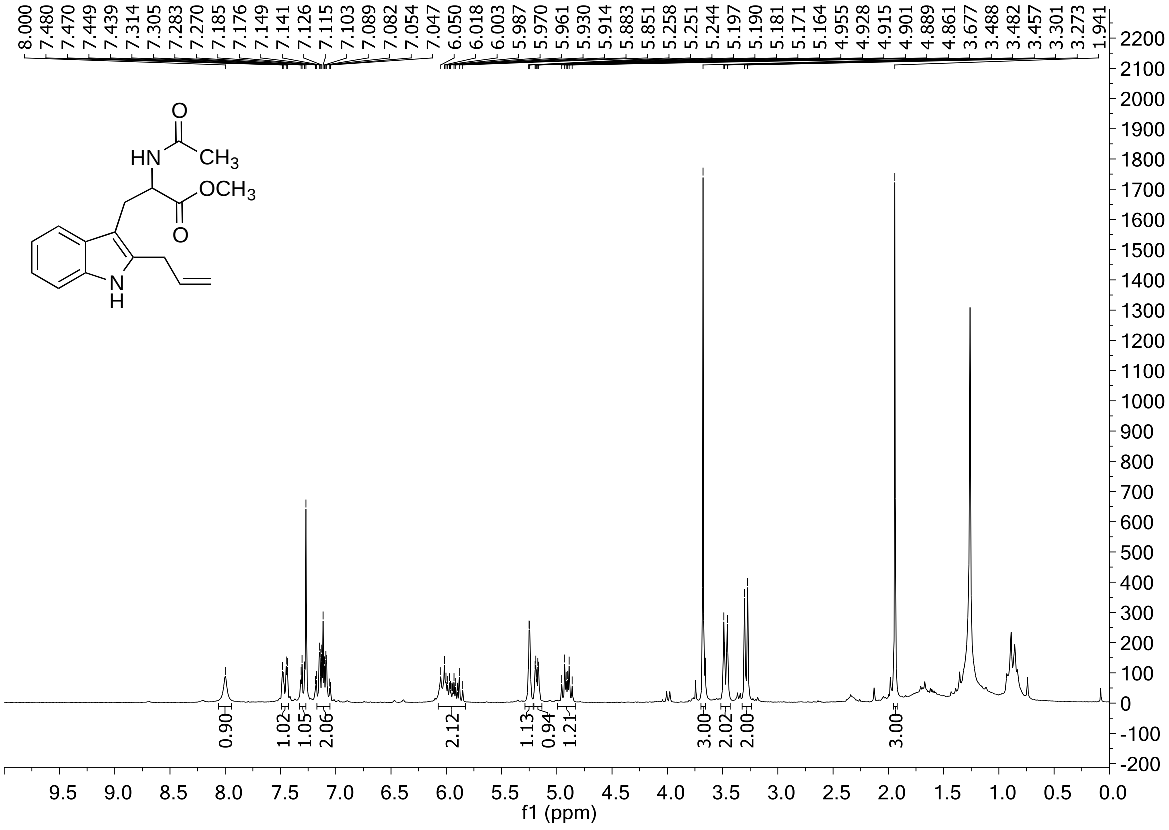

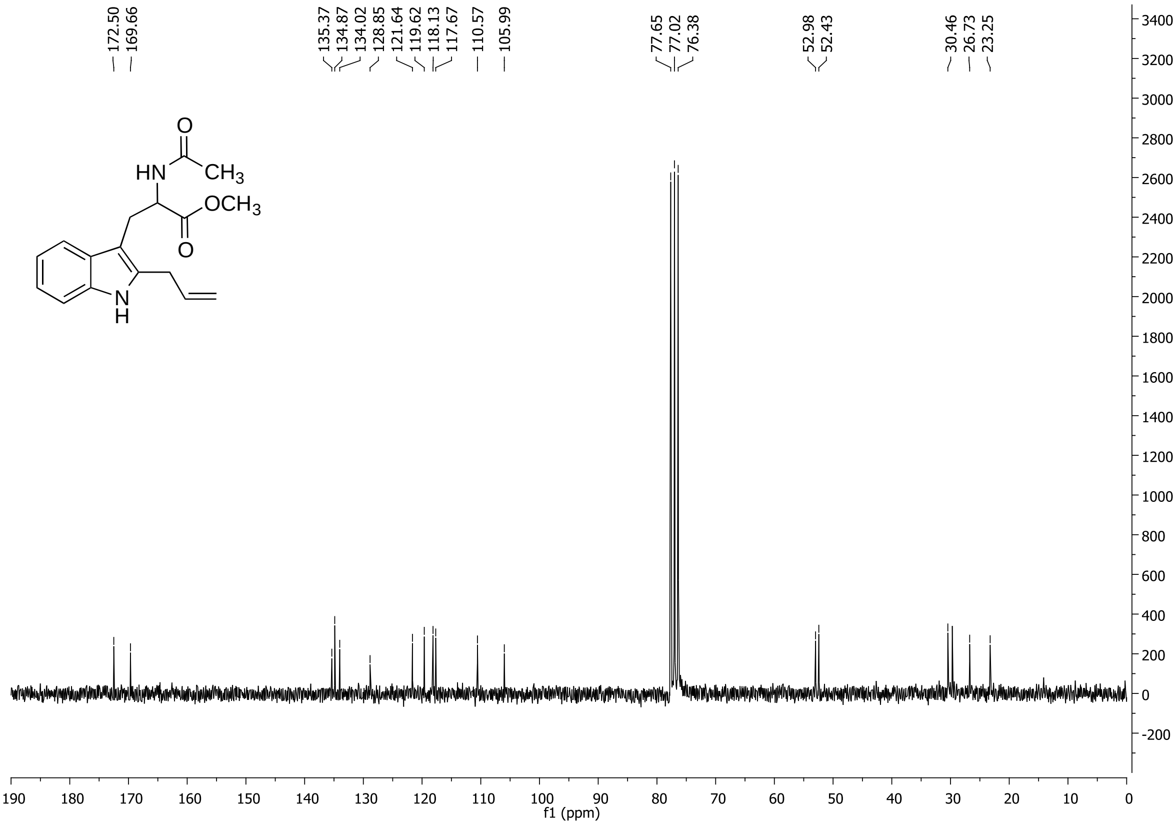

^{1}H and ^{13}C NMR Spectrum of Methyl 2-acetamido-3-(2-(3-methylbut-2-enyl)-1*H*-indol-3-yl)propanoate (3l)

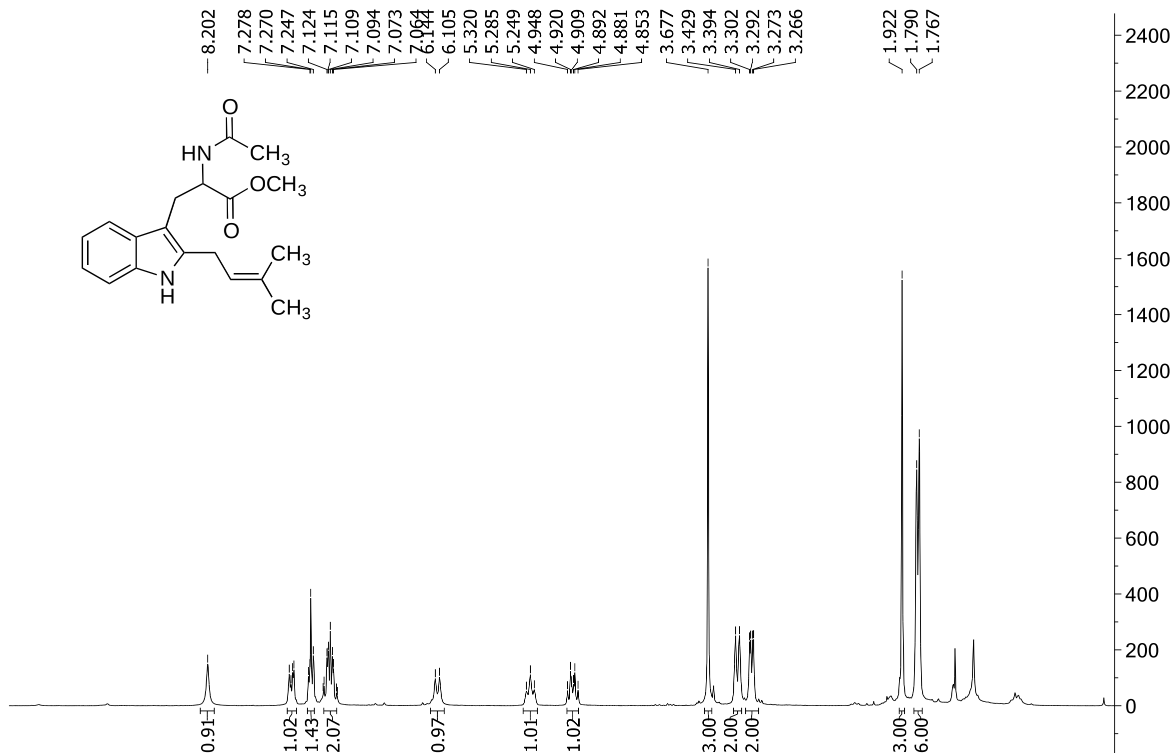

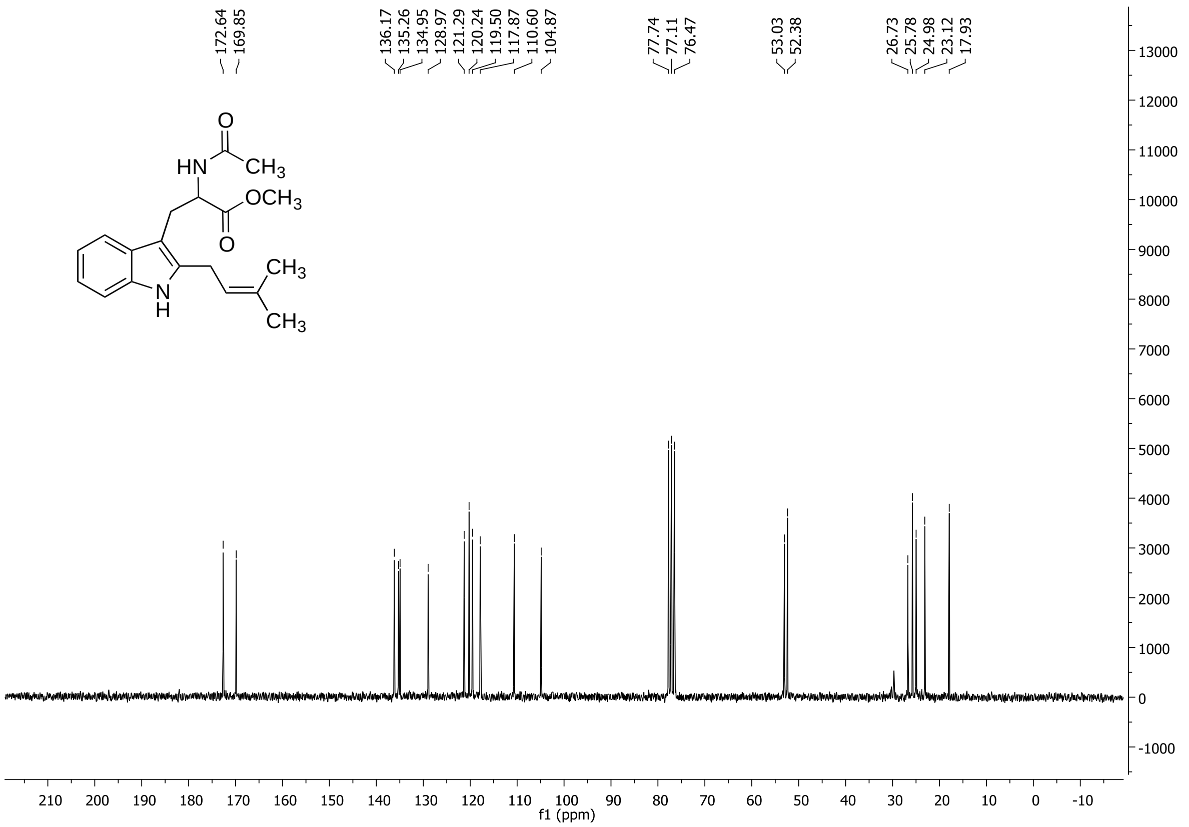

^{1}H and ^{13}C NMR Spectrum of Methyl 2-acetamido-3-(2-((E)-3,7-dimethylocta-2,6-dienyl)-1*H*-indol-3-yl)propanoate (3m)

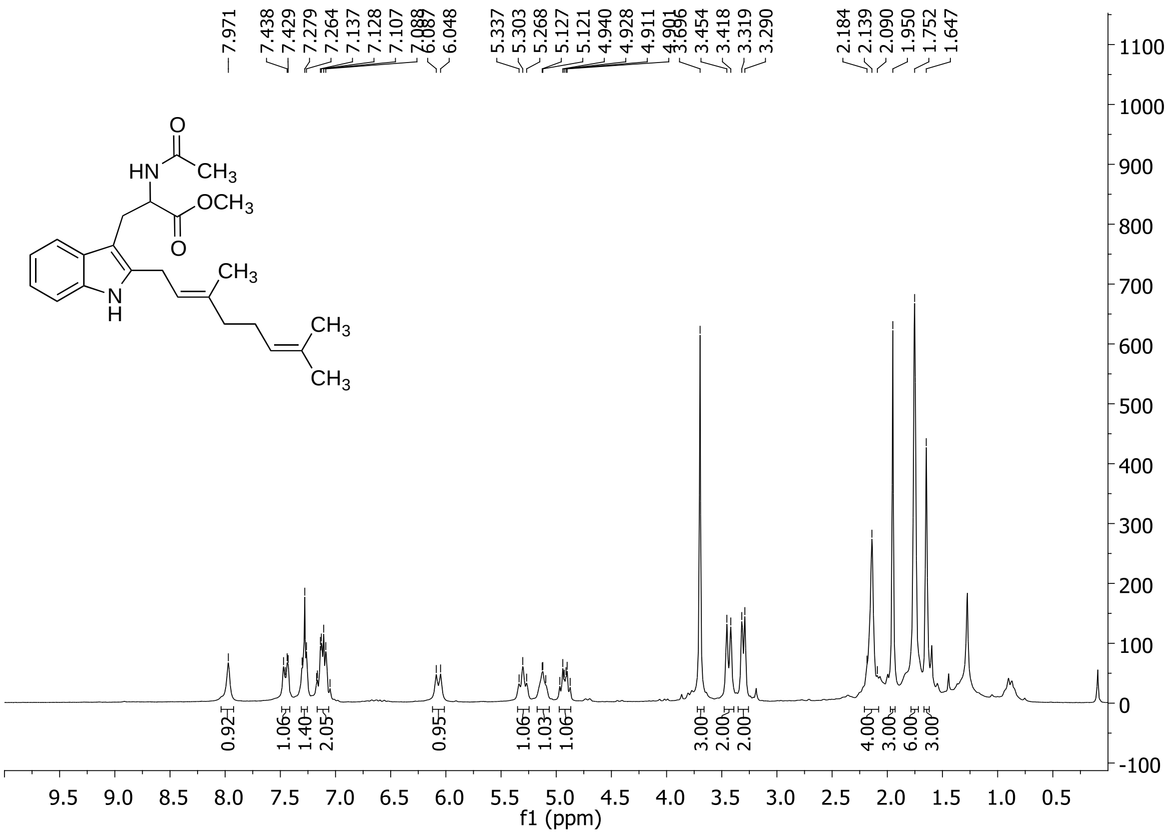

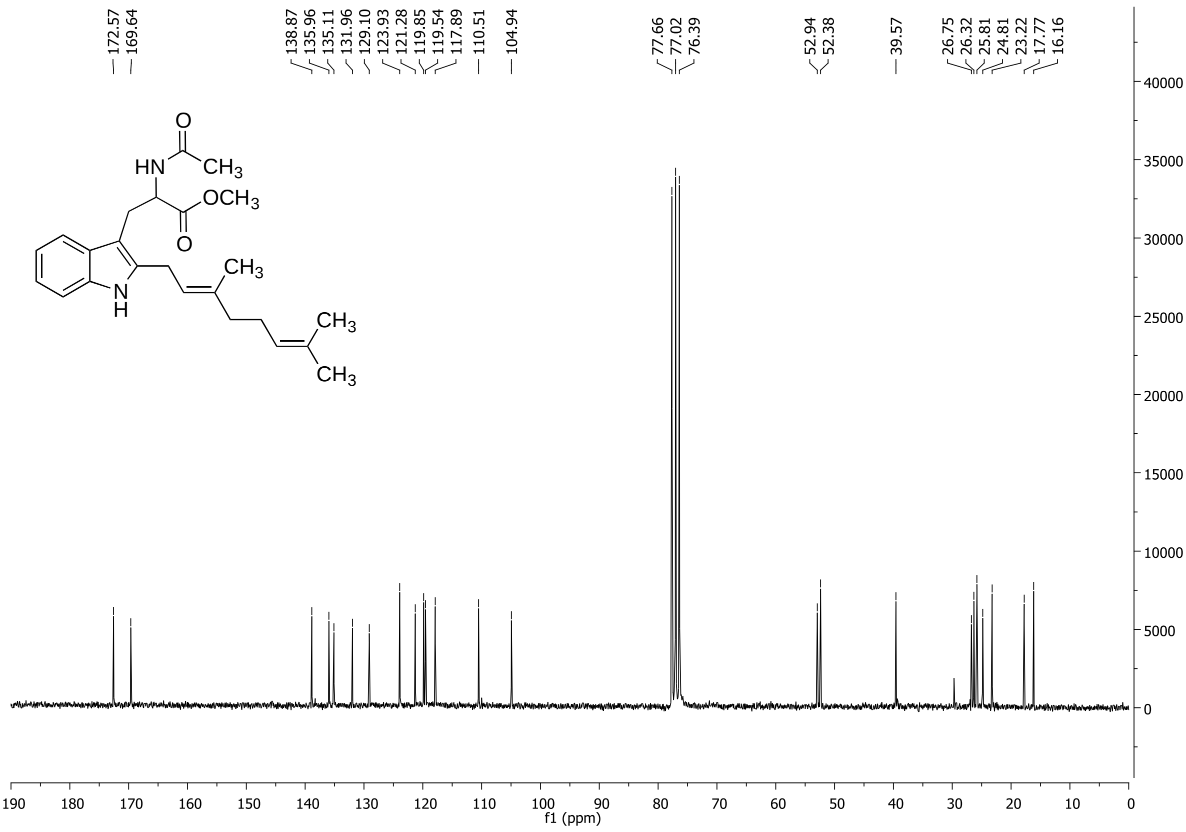

^{1}H and ^{13}C NMR Spectrum of Methyl 2-acetamido-3-(2-(methylthio)-1*H*-indol-3-yl)propanoate (3o)

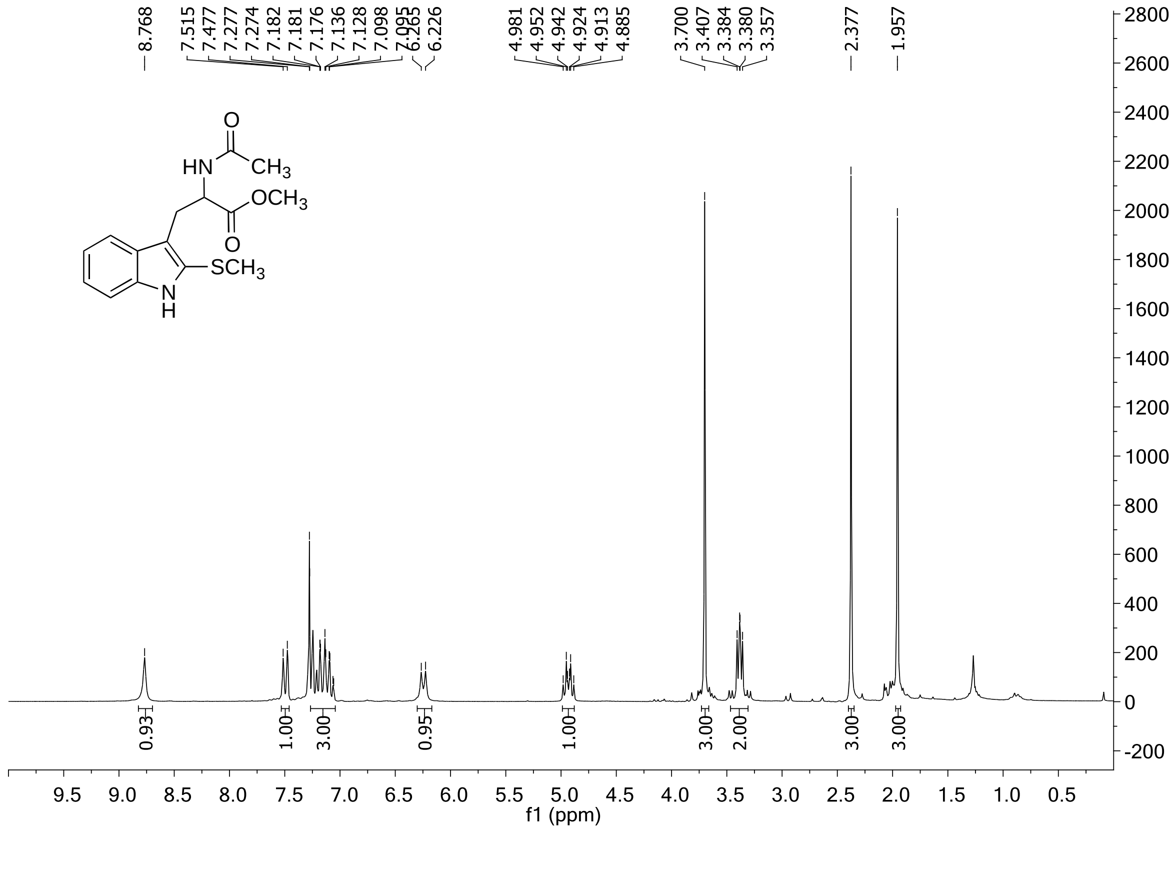

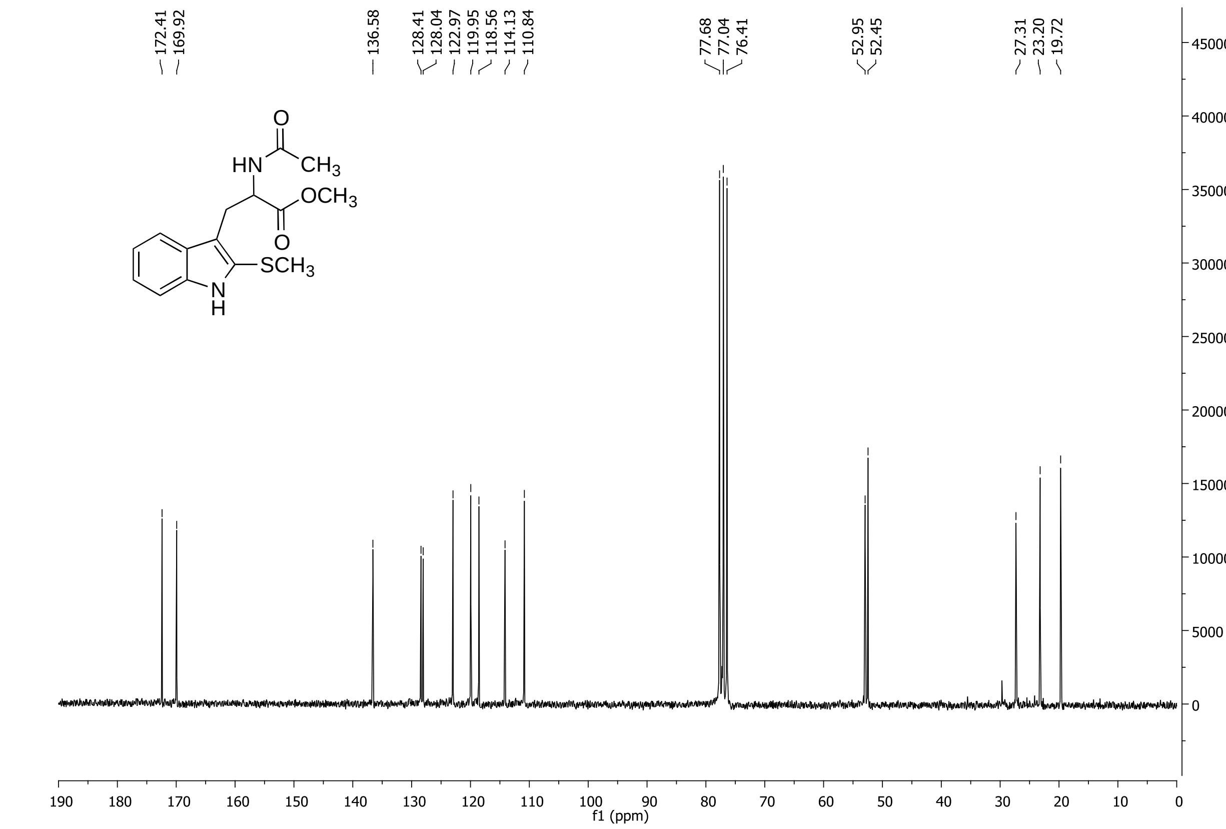

^{1}H and ^{13}C NMR Spectrum of (±)-endo-methyl 1-acetyl-3a-benzyl-1,2,3,3a,8,8a-hexahydropyrrolo[2,3-*b*]indole-2-carboxylate

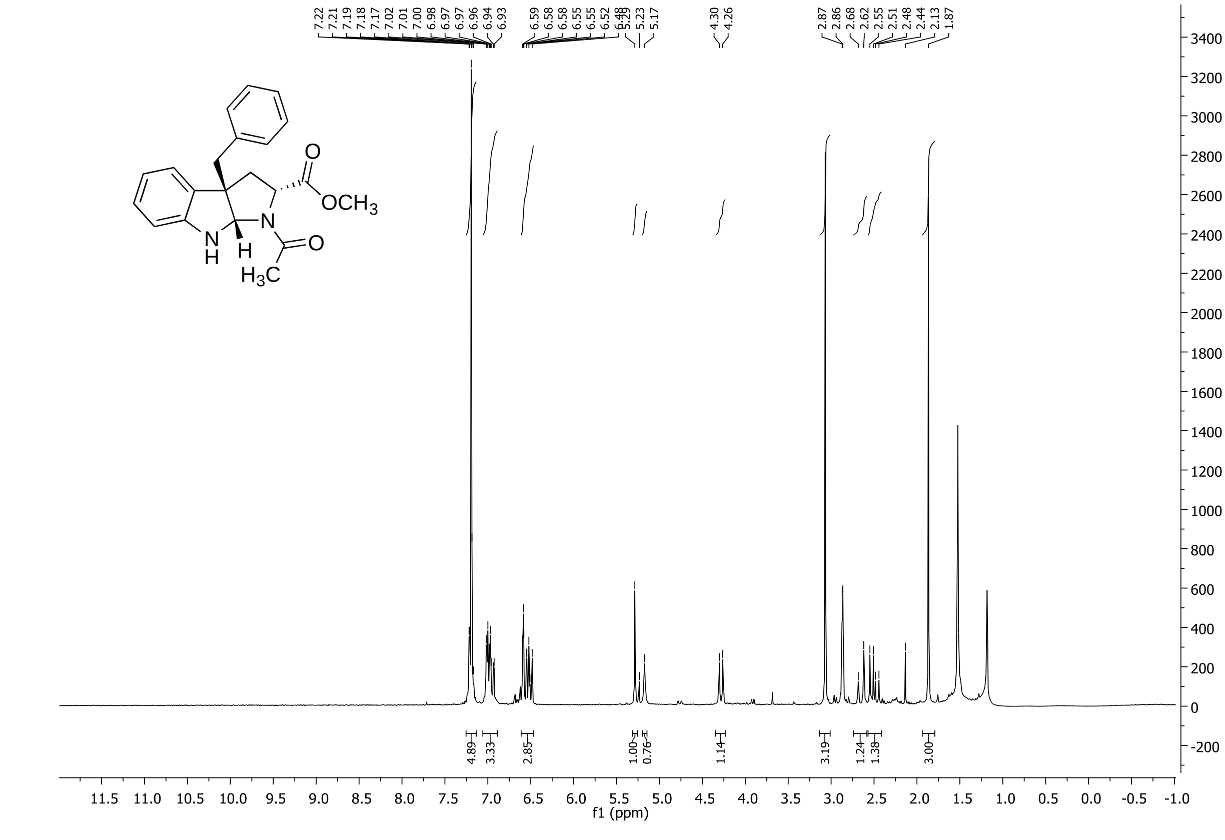

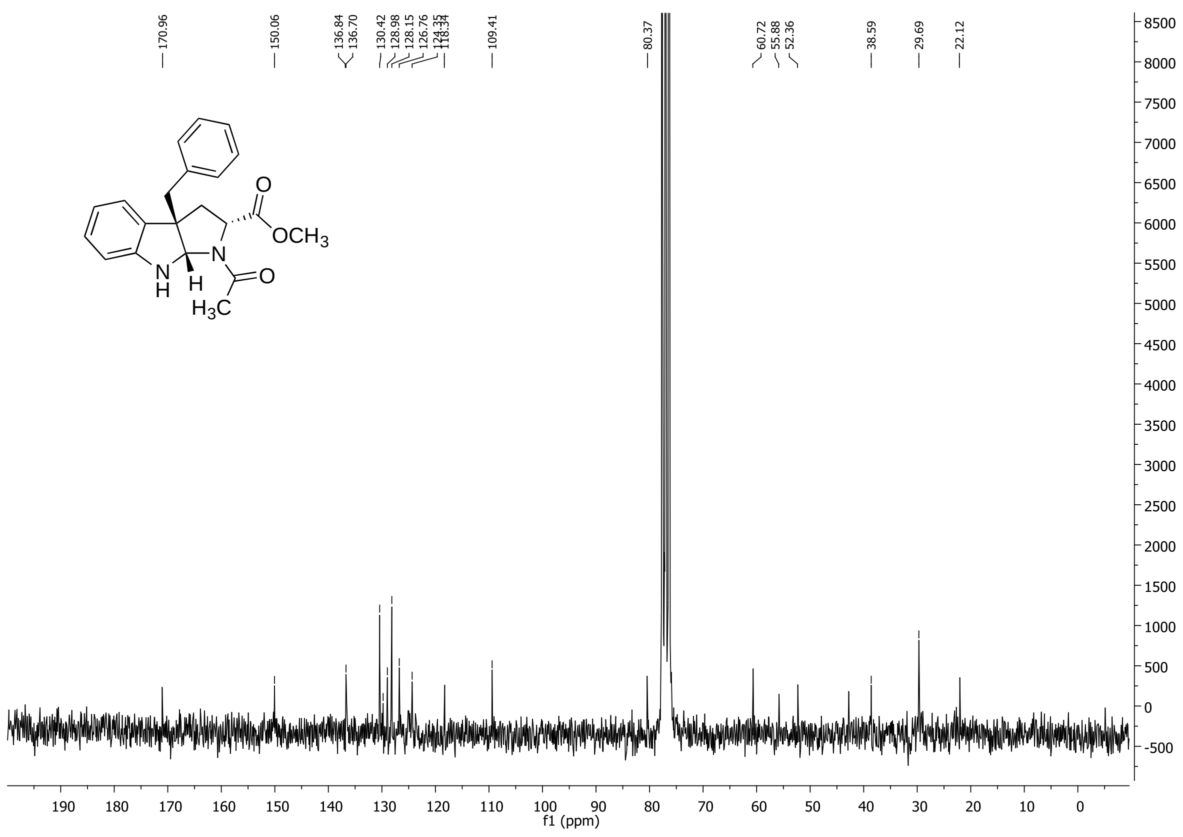

^{1}H and ^{13}C NMR Spectrum of (±)-exo-methyl 1-acetyl-3a-benzyl-1,2,3,3a,8,8a-hexahydropyrrolo[2,3-*b*]indole-2-carboxylate

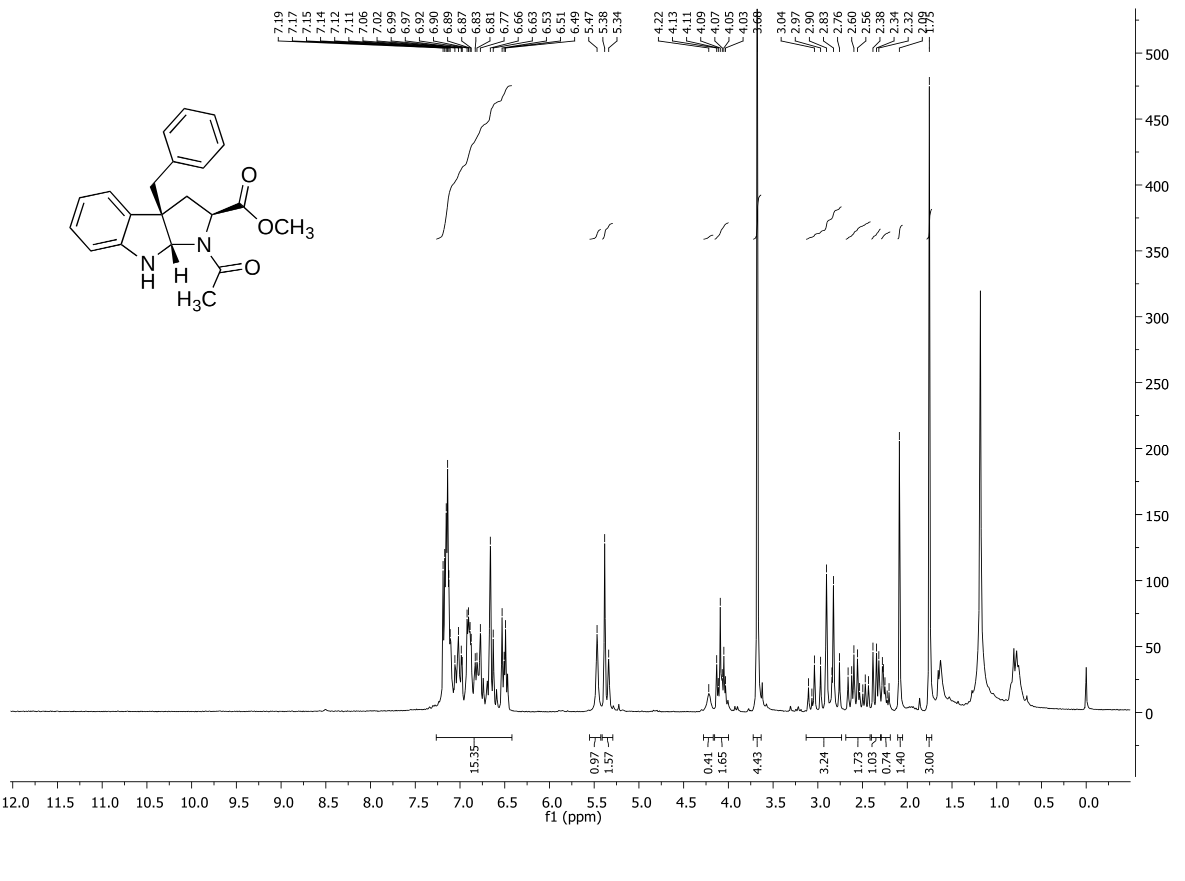

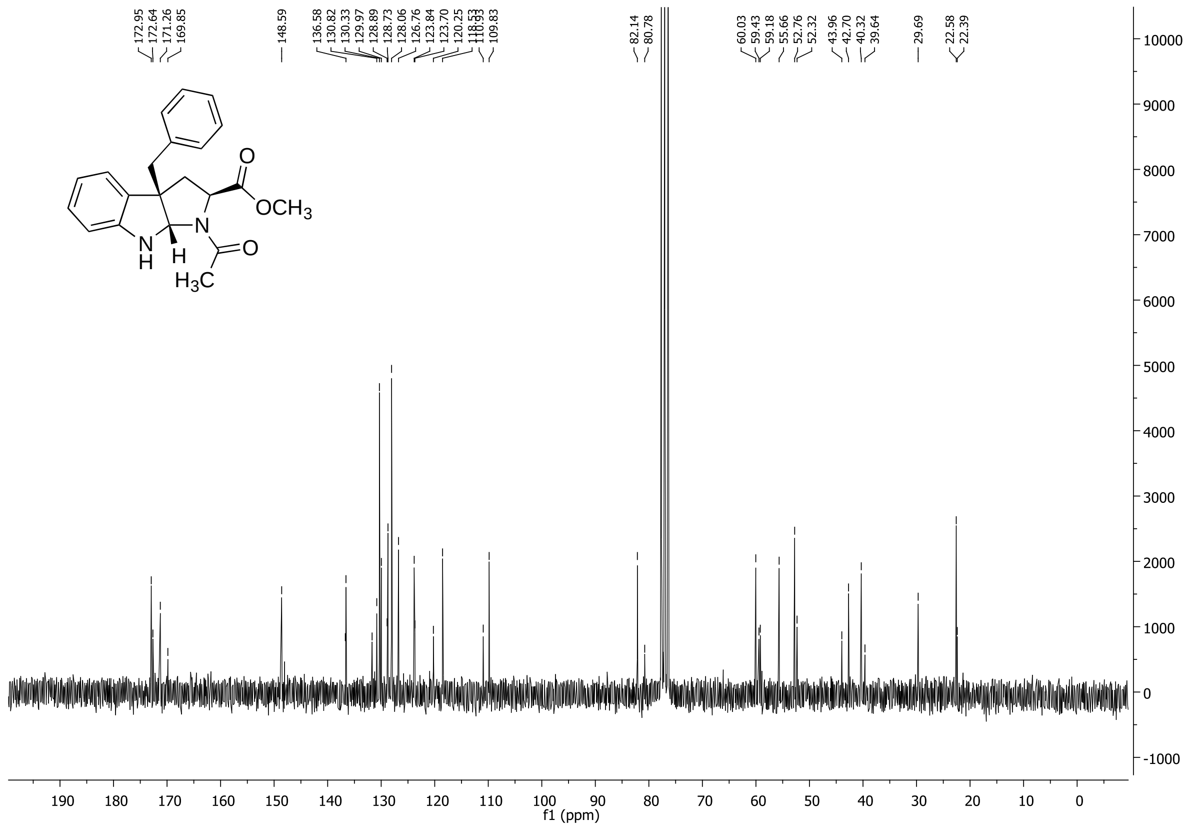
172.95
172.64
171.26
169.85
148.59
136.58
130.82
130.33
129.97
128.89
128.73
128.06
126.76
123.84
123.70
120.25
118.53
110.93
109.83
82.14
80.78
60.03
59.43
59.18
55.66
52.76
52.32
43.96
42.70
40.32
39.64
29.69
22.58
22.39
OCH3
N
H
NH
O
H3C
f1 (ppm)
